# Review and experimental comparison of speckle-tracking algorithms for X-ray phase contrast imaging

**DOI:** 10.1107/S1600577524010117

**Published:** 2025-01-01

**Authors:** Rafael Celestre, Laurène Quénot, Christopher Ninham, Emmanuel Brun, Luca Fardin

**Affiliations:** a Synchrotron SOLEIL L’Orme des Merisiers, Dèpartementale 128 Saint-Aubin France; b Univ. Grenoble Alpes, INSERM, UA7 STROBE, Grenoble, France; Australian Synchrotron, Australia

**Keywords:** X-ray phase contrast, speckle tracking

## Abstract

X-ray speckles have been used in a wide range of experiments, including imaging (and tomography), wavefront sensing, spatial coherence measurements, X-ray photon correlation spectroscopy and ptychography. In this review and experimental comparison, we focus on using X-ray near-field speckle grains as wavefront markers and numerical methods for retrieving the phase information they contain. We present the most common tracking methods, introducing the existing algorithms with their specifications and comparing their performances under various experimental conditions. This comparison includes applications to different types of samples: phantoms for quantitative analysis and complex samples for assessing image quality. Our goal is to unify concepts from several speckle tracking methods using consistent terminology and equation formalism, while keeping the discussion didactic and accessible to a broad audience.

## Abstract 2

This review focuses on low-dose near-field X-ray speckle phase imaging in the differential mode introducing the existing algorithms with their specifications and comparing their performances under various experimental conditions.

## 1. Introduction

Over the past decades, synchrotron X-ray phase contrast imaging (PCI) has become a great tool for the non-destructive study of samples in fields ranging from archaeology to industry and medicine (Cunningham *et al.*, 2014 ▸; Hall, 2021 ▸; Westneat *et al.*, 2008 ▸; Momose, 2020 ▸; Quénot *et al.*, 2022 ▸; Ponchut *et al.*, 2021 ▸). The success of this method, over conventional radiology, is partly due to the possibility of retrieving an image with enhanced edges in samples with low absorption, and enhanced contrast between sub-regions characterized by similar absorption coefficients (Endrizzi, 2018 ▸). The most basic phase contrast technique used at synchrotron light sources is propagation-based imaging (PBI) (Snigirev *et al.*, 1995 ▸; Cloetens *et al.*, 1996 ▸). This in-line phase imaging technique is rather simple to implement, needing illumination with a sufficient degree of spatial coherence, the sample and an imaging film or detector at some distance downstream (Wilkins *et al.*, 1996 ▸). However, experimental conditions and sample restrictions for which quantitative phase information can be extracted limits its applications.

**Figure 1:**
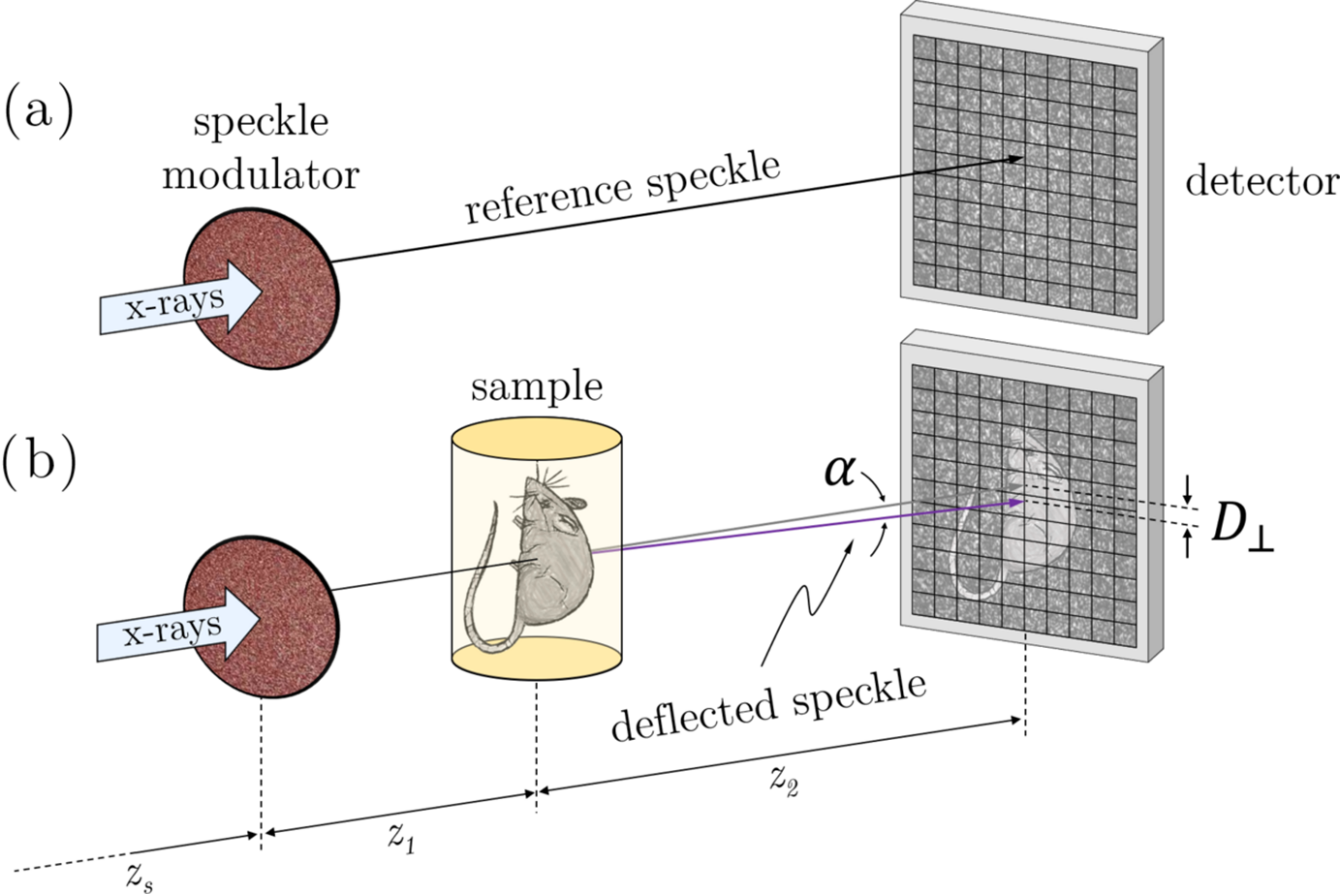
Differential speckle based imaging setup. (*a*) A reference image (set) is recorded with the speckle membrane in the beam; (*b*) the sample is then placed in the beam, distorting the speckle field. A new image (set) is recorded. The object-induced phase can be numerically retrieved by comparing the sample and the reference images. Here *z*_s_ is the distance from the source to the speckle modulator, *z*_1_ from the membrane to the sample, and *z*_2_ the sample-to-detector distance. *D*_⊥_ is the transverse speckle displacement in the detector plane and α the associated deflection angle.

Several other PCI techniques also exist: interferometric methods such as Bonse–Hart interferometry (Bonse & Hart, 1965 ▸); variations of the aforementioned free-space propagation technique (Snigirev *et al.*, 1995 ▸; Cloetens *et al.*, 1996 ▸); crystal analyser diffractometry/X-ray Schlieren method (Förster *et al.*, 1980 ▸); diffraction-grating-based imaging (David *et al.*, 2002 ▸; Weitkamp *et al.*, 2005 ▸); Hartmann (Mercère *et al.*, 2003 ▸; de La Rochefoucauld *et al.*, 2021 ▸) and Shack–Hartmann (Mayo & Sexton, 2004 ▸; Dos Santos Rolo *et al.*, 2018 ▸; Mikhaylov *et al.*, 2020 ▸) type sensors and other forms of wavefront markers tracking, which include speckle tracking. A more complete overview on X-ray phase-contrast imaging techniques is given by Endrizzi (2018 ▸) and Weitkamp *et al.* (2011 ▸); more recently, a review on X-ray in-line imaging by Gureyev *et al.* (2024 ▸) was published containing a very thorough reference list. Speckle based imaging (SBI) – the topic of this work – uses static randomly structured modulators to generate a speckle field – sand paper, filter membranes, metallic powders and coded masks are often used for that purpose (Morgan *et al.*, 2012 ▸; Zdora, 2018 ▸; Berujon *et al.*, 2020*a* ▸; Tian *et al.*, 2020*a* ▸; Qiao *et al.*, 2021 ▸; Quénot *et al.*, 2021*a* ▸; Labriet *et al.*, 2022 ▸; Shi *et al.*, 2023 ▸). A very important characteristic of those speckle grains is that in the near-field regime they preserve shape and size, which permits their use as wavefront markers (Cerbino *et al.*, 2008 ▸; Siano *et al.*, 2021 ▸). In the differential implementation – see §2.1 in Berujon *et al.* (2020*b* ▸) – a sample is introduced into the beam disturbing the reference speckle pattern in the detector. This is represented in Fig. 1 ▸. The object-induced phase can be numerically retrieved by comparing the sample and the reference images. Compared with other techniques, speckle tracking has the advantage of a very simple set-up transferring the experimental complexity to the numerical phase extraction.

In this article, we will present in detail a non-exhaustive list of phase retrieval algorithms for SBI. The algorithms can be sorted into two main categories: explicit tracking based on surveying the local speckle displacements through error minimization or cross-correlation; and implicit tracking based on solving an inverse problem defined by the transport of intensity equation in each pixel. An emergent third group – artificial-intelligence-assisted methods (Qiao *et al.*, 2022 ▸) – will not be considered in this work, because at the time of writing they are still incipient. When discussing the several implementations, we intentionally leave out the dark field calculations, as they are outside the scope of this work. The algorithms are compared under various experimental conditions. Synchrotron and laboratory sources are used to test the results under different degrees of coherence of the illumination. Samples include single material lenses and phantoms, to test for the retrieval of quantitative information, and complex biomedical tissues. For biomedical samples, the results are compared when decreasing the radiation dose, measured as the number of images required for the phase-retrieval. Reducing data acquisition time, while preserving image reconstruction quality, is required to minimize motion artefacts and increase patient comfort in clinical imaging. Furthermore, it helps reducing the radiation dose. These are important factors when foreseeing a clinical translation of the techniques.

## 2. Phase retrieval algorithms: explicit tracking

Explicit tracking methods consist of following the local transverse displacement of the speckle patterns in the detection plane after the sample is inserted, the general idea being that a small subset of pixels centred around an arbitrary pixel (*x*_*i*_, *y*_*j*_) in the sample image(s) and a second set of of pixels in the reference image(s) will be compared (*e.g*. through cross-correlation), calculating the lateral shift *D*_⊥_ from the coordinates (*x*_*i*_, *y*_*j*_) that leads to the best matching between the two sub-sets. This operation is repeated for every pixel (*x*, *y*) of the sample image. Among those algorithms there is a ‘family’ of methods that was derived from the initial X-ray speckle tracking algorithm (Bérujon *et al.*, 2012 ▸) and another one based on a different method called unified modulated pattern analysis (UMPA) (Zanette *et al.*, 2014 ▸; Zdora *et al.*, 2017 ▸). Explicit speckle tracking methods have been extensively discussed by Zdora (2018 ▸) and Berujon *et al.* (2020*b* ▸).

### 2.1. X-ray speckle tracking

The earliest SBI algorithm, called simply X-ray speckle tracking (XST), was first published in 2012 (Bérujon *et al.*, 2012 ▸; Morgan *et al.*, 2012 ▸) and is a generalization of a method developed for attenuation grid imaging by Morgan *et al.* (2011 ▸). XST requires only one pair of sample/reference images, which makes this technique particularly interesting for fast/low-dose applications. The method involves calculating the 2D cross-correlation between two small windows *w* (2*m* + 1 × 2*n* + 1) centred around the pixels (*x*_*i*_, *y*_*j*_) and (*x*_*i*_ + χ, *y*_*j*_ + γ) in the sample (*I*_s_) and reference (*I*_r_) image, respectively. (χ, γ) is a vector in a 2D search interval *W* centred around (0, 0) that is (2*M* + 1) by (2*N* + 1) pixels wide, where *M* and *N* are the largest horizontal and vertical displacements in pixel units to be considered. The transverse displacement *D*_⊥_(*x*_*i*_, *y*_*j*_) of the speckle modulations is given by the parameters χ(*x*_*i*_, *y*_*j*_), γ(*x*_*i*_, *y*_*j*_) ∈*W* which maximize the zero-normalized cross-correlation between the two windows, 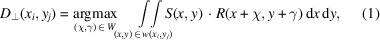
where *S* = 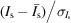 and *R* = 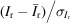, with the bar representing the mean within the window *w* and σ the standard deviation. This template-matching technique is analogous to the 2D Pearson product-moment correlation coefficient. The procedure described in equation (1) is applied to each pixel (*x*_*i*_, *y*_*j*_) within the image or a region of interest (ROI) resulting in 2D maps χ(*x*, *y*) and γ(*x*, *y*) of the horizontal and vertical displacements, respectively. The deflection angles α = (α_*x*_, α_*y*_) and the transverse displacement maps *D*_⊥_(*x*, *y*) = (χ, γ) are related geometrically by
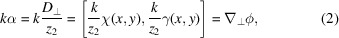
where *k* is the wavenumber and 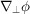 = 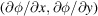 is the phase transverse gradient – this equation is valid under the paraxial approximation. The phase image ϕ(*x*, *y*) can be obtained by numerical integration of the gradients – see §4 for more details. To further increase the angular sensitivity of the technique, several algorithms can be used for sub-pixel peak detection (Fisher & Naidu, 1996 ▸; Sun, 2002 ▸). Despite the procedures in equations (1) and (2) being repeated for each pixel within a ROI containing the sample, this technique has a low spatial resolution as this is inversely proportional to the window size – see Tian *et al.* (2020*a* ▸) for a study on the optimal window size and its influence on the reconstructed phase lateral resolution. This technique is advantageous because it requires only a single pair of sample and reference images, making it particularly suitable for scenarios where reducing data acquisition time is critical, as mentioned in the *Introduction*[Sec sec1]. Additionally, the computational cost is moderate, which enhances its practicality for short-time (Berujon *et al.*, 2015 ▸; Seaberg *et al.*, 2019 ▸) or large-scale applications, *e.g.* tomography (Wang *et al.*, 2015 ▸). However, the lateral resolution of this technique is limited by the size of the speckle grains relative to the detector’s point spread function. Typically, the speckle grains should span a few pixels, which imposes a practical constraint on the achievable resolution.

### 2.2. X-ray speckle vector tracking

The X-ray speckle vector tracking (XSVT) method is a technique which increases the lateral resolution of XST down to a pixel and improves its angular resolution (Berujon & Ziegler, 2015 ▸; Berujon & Ziegler, 2016 ▸). XSVT is a scanning technique that consists of taking *P* image pairs (sample/reference), each pair at a different (randomly chosen) transverse position *p* of the speckle generator. The collected images are organized into 3D stacks, where the index in the third dimension corresponds to the *p*th membrane position. A vector is created from the sample images by reading the intensity values in a given pixel (*x*_*i*_, *y*_*j*_) along the *P* dimension, giving rise to the 1D signal *I*_s_(*x*_*i*_, *y*_*i*_, *p*). Following the same procedure, a set of 1D vectors is then generated from the reference images by reading the intensity values *I*_r_(*x*_*i*_ + χ,*y*_*j*_ + γ, *p*) along *P*. (χ, γ) is a translation vector in a search interval *W*, as in XST.

The 1D vector *I*_s_(*x*_*i*_, *y*_*j*_, *p*) is correlated with each 1D vector *I*_r_(*x*_*i*_ + χ, *y*_*j*_ + γ, *p*), thus creating a 2D matrix of correlation coefficients. The transverse displacement *D*_⊥_(*x*_*i*_, *y*_*j*_) of the speckle vector is retrieved from the coordinate (γ, χ) at which the 2D map of cross-correlation coefficients attains its maximum, 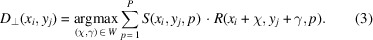
Again, *S* = 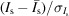 and *R* = 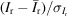 with the bar representing the mean along the *P* direction and σ the standard deviation. The sub-pixel treatments described by Fisher & Naidu (1996 ▸) and Sun (2002 ▸) and phase gradient retrieval from equation (2) are also applied to the transverse displacement *D*_⊥_(*x*_*i*_, *y*_*j*_) obtained by equation (3). As discussed by Qiao *et al.* (2020*a* ▸), the direct implementation of equation (3) is very computer intensive, and vectorizing the operation by using matrix multiplication can speed up the calculations significantly – see §2.1 of Qiao *et al.* (2020*a* ▸). XSVT is a technique that performs best when a large number *P* of image pairs can be acquired, as this improves signal matching and enhances the accuracy of the results. Therefore, in scenarios where the number of image pairs is limited, XSVT may not be the most suitable approach. As long as the window is large enough to contain the matching signal, XSVT maintains high lateral resolution even when the search window size is increased, unlike XST. However, increasing the search window does result in higher computational demands when calculating equation (3). Additionally, XSVT is less suited for applications requiring fast data acquisition, making it impractical for short time-scale experiments.

### 2.3. The XST–XSVT hybrid

Let us consider the same dataset as for a classical XSVT experiment. Instead of calculating the correlation between 1D vectors, a small window *w* = (2*m* + 1 × 2*n* + 1) is considered around the pixel (*x*_*i*_, *y*_*j*_) as in an XST analysis: the 3D vector *I*_s_(*x*, *y*, *p*), (*x*, *y*) ∈ *w*, is obtained. This 3D vector is correlated to a set of 3D vectors *I*_r_(*x* + χ, *y* + γ, *p*), where (*x*, *y*) ∈ *w* and (χ, γ) is a translation vector in the search interval *W* = (2*M* + 1 × 2*N* + 1), as in XST and XSVT. In this case, equation (3) is rewritten as 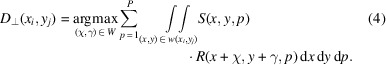
The window *w* is usually kept much smaller than the interval *W* for computational reasons. The same post-treatment dispensed on the XSVT can be applied to the lateral displacement [equation (4)]. This method, called XST–XSVT, was first presented by Berujon & Ziegler (2016 ▸) as a way of reducing the number of exposures *P* of XSVT for imaging applications, while keeping a high angular sensitivity. The cost is a small decrease in lateral resolution due to an increased window size (Tian *et al.*, 2020*b* ▸). This trade-off makes the XST–XSVT hybrid particularly useful when a compromise between number of exposures and resolution is necessary – see Appendix *B*[App appb]. Additionally, the computational cost of this method is very high, making it suitable for cases where the trade-off between reduced exposures and increased computational demand is justified.

### 2.4. Wavelet X-ray speckle vector tracking (WXSVT)

The wavelet-transform-based speckle vector tracking method for X-ray phase imaging (WXSVT) was proposed by Qiao *et al.* (2020*a* ▸) as a way of improving noise robustness and increasing the computational efficiency of the aforementioned XSVT method – Qiao *et al.* (2020*b* ▸) have also produced a wavelet variation of the classical XST algorithm, which is not covered in this work. The data collection is essentially the same as for the XSVT, so are the sample and reference vectors generation, that is, the 1D signal *I*_s_(*x*_*i*_, *y*_*i*_, *p*) and the 3D data set *I*_r_(*x*, *y*, *p*), which is limited by the sampling window *w*.

The WXSVT method relies on applying the discrete wavelet transform (DWT) to both the sample and reference signals, re-writing them in terms of DWT coefficients. The DWT is an orthogonal transform, meaning it preserves the Euclidean distance between the sample *I*_s_(*x*_*i*_, *y*_*i*_, *p*) and the reference *I*_r_(*x*_*m*_, *y*_*n*_, *p*) vectors before and after the transformation – the Euclidean distance calculation is also an operation that can be vectorized, which is beneficial to the computational cost of the calculation. A property of particular interest is that the DWT allows setting cut-off coefficients enabling the reduction of the size of the transformed vector, which behave conceptually similarly to the cut-off frequencies in the Fourier transform. Up to a limit, reducing the number of detail coefficients not only improves computation efficiency but also increases the noise robustness of the method. Alternatively, being more conservative when setting the cut-off coefficients, one can reduce the number of *P* image pairs in the scan while still retaining good phase retrieval, as demonstrated in §3.4 of Qiao *et al.* (2020*a* ▸). This is obviously positive, from the point of view of the dose reduction in the sample. Sub-pixel maxima tracking methods used by XST and XSVT are also applied in the WXSVT method. Once the transverse displacement *D*_⊥_(*x*_*i*_, *y*_*j*_) is obtained, equation (2) can be applied to obtain the phase gradients.

Overall, WXSVT exhibits the same advantages and drawbacks as XSVT, but with improved noise robustness and computational efficiency, particularly when the number of wavelet coefficients is reduced. The computational efficiency of WXSVT is enhanced by setting cut-off coefficients in the wavelet transform, which reduces the size of the transformed vectors.

### 2.5. X-ray speckle scanning and its variations

X-ray speckle scanning (XSS) (Berujon *et al.*, 2012 ▸) and all its variations – see Zdora (2018 ▸) and Berujon *et al.* (2020*b* ▸) – were techniques introduced to achieve a very high angular resolution at the expense of a highly increased number *P* of image pairs (sample/reference) – XSS schemes have been reported to achieve nanoradian angular sensitivity (Wang *et al.*, 2015 ▸; Kashyap *et al.*, 2016 ▸). These techniques require a much more sophisticated setup (long-term stability and reproducibility of motors), with the speckle membrane being scanned with step sizes smaller than the imaging detector pixel size. While these techniques are well adapted for at-wavelength metrology (Berujon *et al.*, 2020*a* ▸), they are ill-suited for low-dose or clinical applications. XSS methods are out of the scope of this work.

### 2.6. Unified modulated pattern analysis

Unified modulated pattern analysis (UMPA) is a data processing scheme that can be applied to data sets compatible with the (W- or XST)–XSVT analysis (Zanette *et al.*, 2014 ▸; Zdora *et al.*, 2017 ▸). This method is based on modelling the X-ray interactions with the sample into three distinctive effects: (i) absorption in the sample reducing the intensity transmission 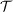; (ii) refraction causing the distortion of the speckle field *D*_⊥_(*x*, *y*) = (χ, γ); and (iii) scattering (dark field) of unresolved features in the sample decreasing the visibility of the speckle fields 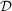 (Zanette *et al.*, 2014 ▸; see also the supplementary material therein). The X-ray beam intensity in the presence of the sample at the detector plane can be modelled as
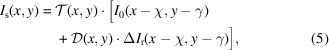
where *I*_r_(*x*, *y*) = *I*_0_(*x*, *y*) + Δ*I*_r_(*x*, *y*) is the reference signal decomposed into an average-valued signal *I*_0_(*x*, *y*) and the fluctuations around this constant value Δ*I*_r_(*x*, *y*). The UMPA method obtains the signals 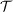, 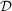 and the displacement maps (χ, γ) by minimizing the sum of squared differences of the cost function, 
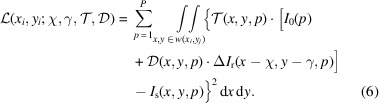
Both 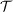 and 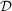 can be analytically obtained from equation (6) by simultaneously solving 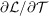 = 0 and 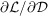 = 0 – see equations 5 and 6 in the supplemental material of Zanette *et al.* (2014 ▸) for the formulations. Much like the XST–XSVT method, the signals *I*_r_ and *I*_s_ are also limited by the window functions *w* and *W* centred around the pixel (*x*_*i*_, *y*_*i*_),
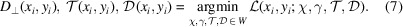
Sub-pixel algorithms are also employed for increasing the angular sensitivity in *D*_⊥_ and the final conversion from transverse displacement χ and γ into phase gradients is done using equation (2). A very informative overview of the UMPA numeric implementation as well as latest developments are given by De Marco *et al.* (2023 ▸). UMPA provides a detailed analysis by separately modelling absorption, refraction and scattering effects. However, it shares a significant drawback with XST–XSVT in terms of computational cost and lateral resolution worsening for increased window size – see Appendix *B*[App appb]. The high computational demands of UMPA arise from the complexity of the model and the need to process and optimize multiple components simultaneously. While UMPA is valuable for comprehensive modelling of X-ray interactions, its computational intensity should be considered when resources are limited.

### 2.7. Comments on the explicit tracking methods

The methods presented here along with their technical challenges and algorithmic implementations are described in more detail elsewhere (Zdora, 2018 ▸; Berujon *et al.*, 2020*a* ▸; Berujon *et al.*, 2020*b* ▸; Qiao *et al.*, 2020*a* ▸). Despite advancements in optimization, a common limitation across these techniques is their moderate to high computational cost. This challenge stems from several factors:

XST: involves calculating 2D cross-correlations between sample and reference images around each pixel. This process can be computationally intensive, particularly with large images or extensive search windows.

XSVT: improves resolution by correlating 1D vectors from multiple image pairs, but it requires significant computational resources to process the 3D stacks of data.

XST–XSVT hybrid: combines elements of XST and XSVT, providing enhancements in sensitivity as a trade-off to lateral resolution. However, it also increases computational complexity due to the dual processing requirements.

WXSVT: builds on XSVT by incorporating wavelet transforms to improve noise robustness and efficiency. The computational benefits are most apparent when reducing the number of wavelet coefficients, but the method still demands considerable computational power, especially with many image pairs.

UMPA: offers detailed modelling by separating absorption, refraction and scattering effects, which enhances accuracy. However, the method involves high computational demands due to the need for complex simultaneous optimization of multiple parameters.

In applications such as tomography, where each projection undergoes the full phase-retrieval pipeline, these methods can become impractically slow. Efficient computing resources and parallelized code are essential to manage the computational load and reduce processing times for large datasets or numerous projection.

## 3. Phase retrieval algorithms: implicit tracking

Implicit speckle tracking is the general name given to a family of techniques based on solving the transport of intensity equation (TIE) under different assumptions related to the sample. For a monochromatic scalar paraxial electromagnetic wave with intensity *I*(*x*, *y*, *z*) propagating along the *z*-direction (optical axis),
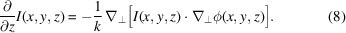
Equation (8) (TIE) can be derived from the paraxial Helmholtz equation, meaning it is subjected to the same assumptions used in the scalar diffraction theory and the paraxial approximation in optics (Paganin, 2006 ▸; Zuo *et al.*, 2020 ▸); furthermore, it is relevant to mention that the TIE is an expression of energy conservation, relating the axial intensity derivative (left-handed side) with the total energy variation in the transverse plane (right-handed side) (Zuo *et al.*, 2020 ▸). A very complete tutorial on the TIE and its applications together with several methods for deriving equation (8) and algorithms for solving it are presented by Zuo *et al.* (2020 ▸).

Equation (8) can be approximated by its finite-difference form for an X-ray beam flowing through a sufficiently small distance (*i.e.* high Fresnel number) *d*_*z*_, 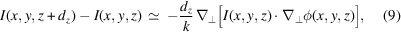
which is also known as the X-ray Fokker–Planck equation (F–PE) for paraxial imaging (Paganin & Morgan, 2019 ▸). This is the form of the TIE used for deriving the implicit methods presented here. A generalization of the F–PE accounting for diffusive paraxial energy transport – similar to what is conveyed by 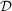 in the UMPA method in §2.6 – has been proposed by Paganin & Morgan (2019 ▸). The use of the F–PE for SBI and retrieval of dark-field signal has been proposed by Pavlov *et al.* (2020*b* ▸) and Pavlov *et al.* (2021 ▸).

### 3.1. Optical flow algorithm

In 2018, Paganin *et al.* (2018 ▸) proposed applying the finite difference version of the TIE [equation (9)] adapted to the SBI problem assuming a perfectly transparent sample (ideal phase element) distorting the speckle field under a quasi-coherent illumination (Gureyev *et al.*, 2006 ▸), 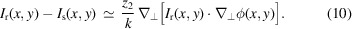
The developments that follow, including the equations, were taken from Paganin *et al.* (2018 ▸). Applying the chain-rule on the right-handed side of equation (10), one obtains two terms: 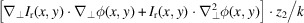. The first term is often referred to as the ‘prism’ term and is responsible for laterally shifting the image at *z* + *d*_*z*_, while the second term is called the ‘lensing-term’ (de-)concentrating light. The lensing-term (or Laplacian term) corresponds to the variations of intensity known as phase contrast. The term ∇_⊥_ϕ(*x*, *y*) in equation (10) can be replaced by the relation expressed in equation (2), resulting in 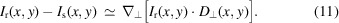
It is important to recall that *D*_⊥_(*x*, *y*) is of vectorial nature and is composed of the scalar fields χ(*x*, *y*) in the *x*-direction and γ(*x*, *y*) in the *y*-direction. In order to facilitate solving equation (11), the multiplicative term *I*_r_(*x*, *y*)·*D*_⊥_(*x*, *y*) is approximated by the gradient of an auxiliary scalar function ∇_⊥_Λ(*x*, *y*) – see Paganin *et al.* (2018 ▸) for entailing approximations leading to equation (3) from that publication. This manipulation allows equation (11) to be rewritten as a Poisson-type equation,
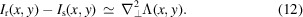
A simple way to solve equation (12) and extract the lateral displacements *D*_⊥_(*x*, *y*) is by using Fourier direct and inverse transforms (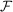 and 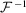) as follows,
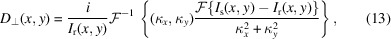
where *i* is the imaginary unity and (κ_*x*_, κ_*y*_) are the coordinates in the Fourier space. After that, equation (2) is used to retrieve the phase gradients. Equation (13) is the result of applying the Fourier derivative theorem.

Solving the TIE equation under the assumptions leading to equation (13), *i.e.* using the optical flow (OF) method, has two main advantages: (i) numerically solving equation (13) is very fast and computationally efficient – only two fast Fourier transforms (FFT) are necessary; and (ii) only one sample/reference pair is required, which makes the OF very dose-efficient.

However, this method assumes a non-absorbing sample, which is rarely the case for clinical samples with dense structures. Since the absorption signal cannot be entirely removed from the dataset, the phase signal retrieved by OF includes filtered absorption effects, making it impossible to isolate the phase signal alone. The absorption due to the sample can be partially estimated and corrected for by blurring *I*_s_(*x*, *y*) and *I*_r_(*x*, *y*), to reduce the speckle modulation, and by calculating the ratio of the resulting images. A drawback of the OF method is that equation (13) approaches a singularity as 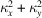

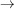
 0. This can be tackled by masking out the singular point (Paganin *et al.*, 2018 ▸) or by applying a Gaussian-shaped high-pass filter in the signal (Rouge-Labriet *et al.*, 2021*a* ▸). Filtering out lower frequencies is detrimental to applications interested in slow varying changes in the object, *e.g.* metrology (Berujon *et al.*, 2020*a* ▸); generally, high-pass filters are tolerated for imaging applications.

### 3.2. Single material object speckle tracking

The single material object speckle tracking (SMOST) method (Pavlov *et al.*, 2020*a* ▸) was conceived to extend the OF algorithm to monomorphous absorbing samples (Gureyev *et al.*, 2015 ▸). It starts by manipulating equation (10) into
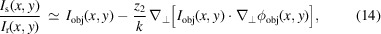
where *I*_obj_(*x*, *y*) and ϕ_obj_(*x*, *y*) are the sample transmitted intensity and phase – refer to Pavlov *et al.* (2020*a* ▸) for a derivation of equation (14). This sample has projected thickness along the optical axis given by Δ_*z*_(*x*, *y*) and is composed of a material with a complex index of refraction *n* = 1 − δ + *i*·β. The values for δ (index of refraction decrement) and β (absorptive part) can be found in tables (Hubbell *et al.*, 1975 ▸; Henke *et al.*, 1993 ▸) and computer libraries (Brunetti *et al.*, 2004 ▸). If the sample is well approximated by a thin-single-material object (Paganin, 2006 ▸), it induces a phase-shift ϕ_obj_(*x*, *y*) = −*k*δΔ_*z*_(*x*, *y*) and an intensity transmission 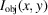 = 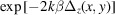. Substituting these two expressions into equation (17) leads to
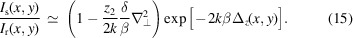
Equation (15) can be solved for Δ_*z*_(*x*, *y*) by
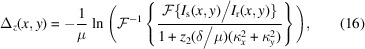
where μ = 2*k*β. Due to the Fourier transforms involved, filtering is sometimes employed to reduce low-frequency artefacts. Unlike the previous methods that retrieve the transverse displacement *D*_⊥_(*x*, *y*), SMOST retrieves directly the sample thickness in projection approximation Δ_*z*_(*x*, *y*), which is linearly proportional to the phase shift ϕ(*x*, *y*) for fixed energy. As pointed out by Pavlov *et al.* (2020*a* ▸), equation (16) bears strong resemblance with the method of Paganin *et al.* (2002 ▸) for propagation-based X-ray phase-contrast imaging.

### 3.3. Low coherence system algorithm

The assumptions used in deriving the OF algorithm are quite restrictive and not always attainable for samples of interest or under common experimental conditions. Although SMOST already handles absorbing samples, it still relies on a coherent illumination – this can be seen by the Laplacian dependency of the phase in equation (15). Relaxation of that hypothesis in OF or SMOST, namely the necessity of transparent sample and high coherence, leads to the low coherence system algorithm (LCS) (Quénot *et al.*, 2021*b* ▸; Quénot *et al.*, 2021*c* ▸). To begin with, the sample image in the presence of the speckle field *I*_s_ can be corrected by an additional ‘loss term’ *I*_obj_ to account for the attenuation due to the sample – this intensity transmission term has been alluded to in the previous section. Equation (11) can, then, be re-written as
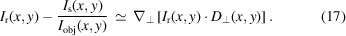
The manipulation in equation (17) allows the TIE framework to be extended into absorbing samples. We now expand the right-handed side of equation (17) using the chain rule,
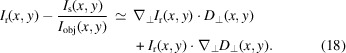
We recall that 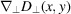 = 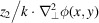. Quénot *et al.* (2021*c* ▸) state that, if the system has low coherence, the Laplacian term could be neglected as the the interference fringes could not be resolved. This leads to 
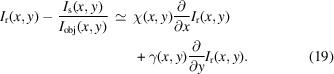
Note that ∇_⊥_*I*_r_ goes to 0 in the absence of a speckle field with high visibility and small speckle grains: in this case, no (good) phase retrieval can be performed. Equation (19) has three unknown variables: the two transverse displacement arrays χ and γ; and the loss term *I*_obj_(*x*, *y*), hence a system with *p* ∈ {1⋯*P*}, *P* ≥ 3, equations can be solved for those three aforementioned variables, 
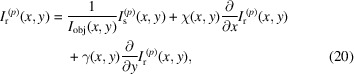
where each equation with a superscript *p* corresponds to a sample/reference image pair, each pair at a different (randomly chosen) transverse position *p* of the speckle generator. A system with *P* > 3 is said to be over-determined and is usually solved by applying a pixel-wise least-squared algorithm to it as proposed by Pavlov *et al.* (2020*b* ▸). This processing permits sub-pixel displacements of the speckle pattern to be tracked (Quénot *et al.*, 2021*c* ▸). Although equation (19) assumes an incoherent illumination, if the sample is indeed illuminated by a coherent beam, the phase contrast fringes that arise due to the Laplacian term in equation (18) will be attributed to the loss term *I*_obj_(*x*, *y*) when solving the system in equation (20). Once the vectors χ and γ are found, equation (2) relates them to the phase gradients.

### 3.4. X-ray multi-modal intrinsic-speckle-tracking

The MIST family of methods is based on the Fokker–Planck equation, a generalization of the TIE (Paganin & Morgan, 2019 ▸), 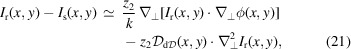
where 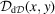 is the diffusive (dark field) term. Comparing the F–PE [equation (21)] and the TIE [equation (10)] allows it to be said that the MIST methods are a natural expansion of the OF-based ones. Solving equation (21) can be done by applying the same assumptions used for SMOST for the first term in the right-hand side of the F–PE and several other assumptions for expanding the second term (Pavlov *et al.*, 2020*b* ▸; Pavlov *et al.*, 2021 ▸; Alloo *et al.*, 2022 ▸; Alloo *et al.*, 2023 ▸). This review does not cover the calculation or applications of the 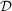 signal and therefore the MIST family will not be considered here.

### 3.5. Comments on the implicit tracking methods

Overall, while these implicit methods provide solutions that are less computationally intensive compared with explicit methods and require fewer image acquisitions, their assumptions can limit their applications. They are particularly attractive for biological imaging and are good candidates for imaging specimens in 3D (tomography). However, while they are very accurate for sub-pixel displacements, they may not track large speckle displacements effectively:

OF: is efficient for phase retrieval with minimal computational overhead, requiring only two FFTs. Despite being less computationally intensive, it assumes a non-absorbing sample, a condition rarely met experimentally: this is expected to introduce a contamination of the filtered absorption in the retrieved phase. The method’s assumptions limit therefore its applicability.

SMOST: extends OF to handle absorbing samples by estimating sample thickness directly. It is advantageous in dealing with absorbing materials but relies on coherent illumination and assumes a monomorphous sample. These assumptions can limit its effectiveness for samples with complex or varying absorption properties. Additionally, like OF, SMOST may struggle with large speckle displacements.

LCS: relaxes the high coherence requirement and handles absorbing samples by introducing a loss term to the TIE framework (like SMOST did). LCS may struggle with very low visibility speckles or high contrast fringes, which can lead to challenges in accurately retrieving phase information. Large speckle displacements can also be challenging for this method: notice that equation (19) effectively corresponds to a first-order Taylor expansion of *I*_r_ around (*x*, *y*).

The implicit methods are described in depth in the literature (Paganin *et al.*, 2018 ▸; Pavlov *et al.*, 2020*a* ▸; Quénot *et al.*, 2021*c* ▸; Rouge-Labriet *et al.*, 2021*b* ▸; Quénot *et al.*, 2022 ▸).

## 4. Integration methods

With the exception of the SMOST method, all algorithms presented here retrieve the transverse deflection maps *D*(*x*, *y*) = (χ, γ). Phase images are obtained by integrating numerically the gradients in equation (2), a problem which, in the presence of noise, is ill-posed (Ettl *et al.*, 2008 ▸; Huang *et al.*, 2015 ▸). Several two-dimensional integration methods for surface reconstruction from gradient data were developed by Frankot & Chellappa (1988 ▸), Arnison *et al.* (2004 ▸) and Kottler *et al.* (2007 ▸). In this work, Frankot Chellappa’s integration method (FC) is used to compare the different phase retrieval algorithms,
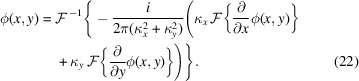
FC is a Fourier-based solver and assumes periodic boundary conditions, which are often not satisfied, especially when the sample extends beyond the edges of the field of view. To avoid artefacts in the integrated phase, an anti-symmetrization of χ and γ was implemented, as suggested by Bon *et al.* (2012 ▸).

## 5. Numerical implementation

All the phase retrieval algorithms presented here, together with several numerical integration methods, are conveniently centralized in open-access GitHub repositories.^1^ Most of the presented algorithms are available through a Python library called POPCORN (POst-processing Phase COntrast and spectRal X-ray imagiNg) (POPCORN, 2021 ▸). The UMPA code used in this work is taken from UMPA (2017 ▸) – an updated version is available at Refactored UMPA (2022 ▸). The WXSVT implementation is taken from WXSVT (2020 ▸).

## 6. Methods: samples description and experimental parameters

The phase retrieval schemes, previously presented, were applied to four different sample groups under diverse experimental conditions. The relevant experimental parameters are summarized in Table 1 ▸.

The first samples were cylindrical nylon wires of 140 µm and 200 µm diameter, which were measured on the ID17 beamline at ESRF. Nylon wires are a common phantom for X-ray phase imaging and were chosen to benchmark quantitatively the speckle tracking algorithms as they offer a simple analytical model for the phase gradient. The second set is composed of three bi-concave parabolic X-ray lenses made out of beryllium (Lengeler *et al.*, 2004 ▸), which are typical samples for metrology measurements. These focusing lenses have different characteristic radii (50 µm, 500 µm and 5000 µm) and were chosen due to their varying strength of phase modulation: the smaller the radius, the stronger the phase variation leading to larger speckle displacements. The lenses were measured on the BM05 beamline at ESRF. A mouse knee was chosen for a qualitative evaluation of the algorithms when applied to biomedical samples. Biomedical samples are characterized by high spatial frequencies, discontinuities and interfaces between different materials, which should be correctly retrieved while reducing the radiation dose. These measurements were also performed at the ID17 beamline. The experiments described so far were performed on a synchrotron source with monochromatic illumination achieved with a Si(111) double-crystal monochromator (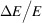 ≃ 10^−4^). The X-ray source for the synchrotron experiments is very small (a few tens of micrometres in the horizontal and less than that vertically) and is placed rather far from the speckle generator (40 m for BM05 and 138 m for ID17). Since the beam is not conditioned by any collimating or refocusing optics, it is safe to assume the illumination on the membrane has an elevated degree of spatial coherence – this is directly a consequence of the van-Cittert–Zernike theorem (Geloni *et al.*, 2008 ▸; Gureyev *et al.*, 2017 ▸). Finally, the last sample – a headless domestic fly – was measured on a micro-focus laboratory X-ray source with a broad spectrum (tungsten anode at 40 kVp) in order to test the algorithms on yet another complex sample with small features but under a low-coherence illumination. The measurements were performed on an adapted EasyTom XL tomographic set-up from RX solutions at the SIMaP (University Grenoble Alpes, France).

## 7. Results: algorithms comparison

In this section we present an experimental comparison of various speckle tracking methods. To ensure consistency, we used the same parameters when comparing the same sample and technique. For example, within the same section, XSVT and WXSVT were always compared using the same window size, and similarly for XST–XSVT and UMPA. The OF and SMOST reconstructions were performed for each individual image pair and merged using the median of several reconstructions. In the LCS method, the linear system was solved using QR (Golub & Van Loan, 2013 ▸) decomposition. Detailed reconstruction parameters can be found in Appendix *A*[App appa].

### 7.1. Nylon wires: phantom imaging and quantitative analysis

**Figure 2:**
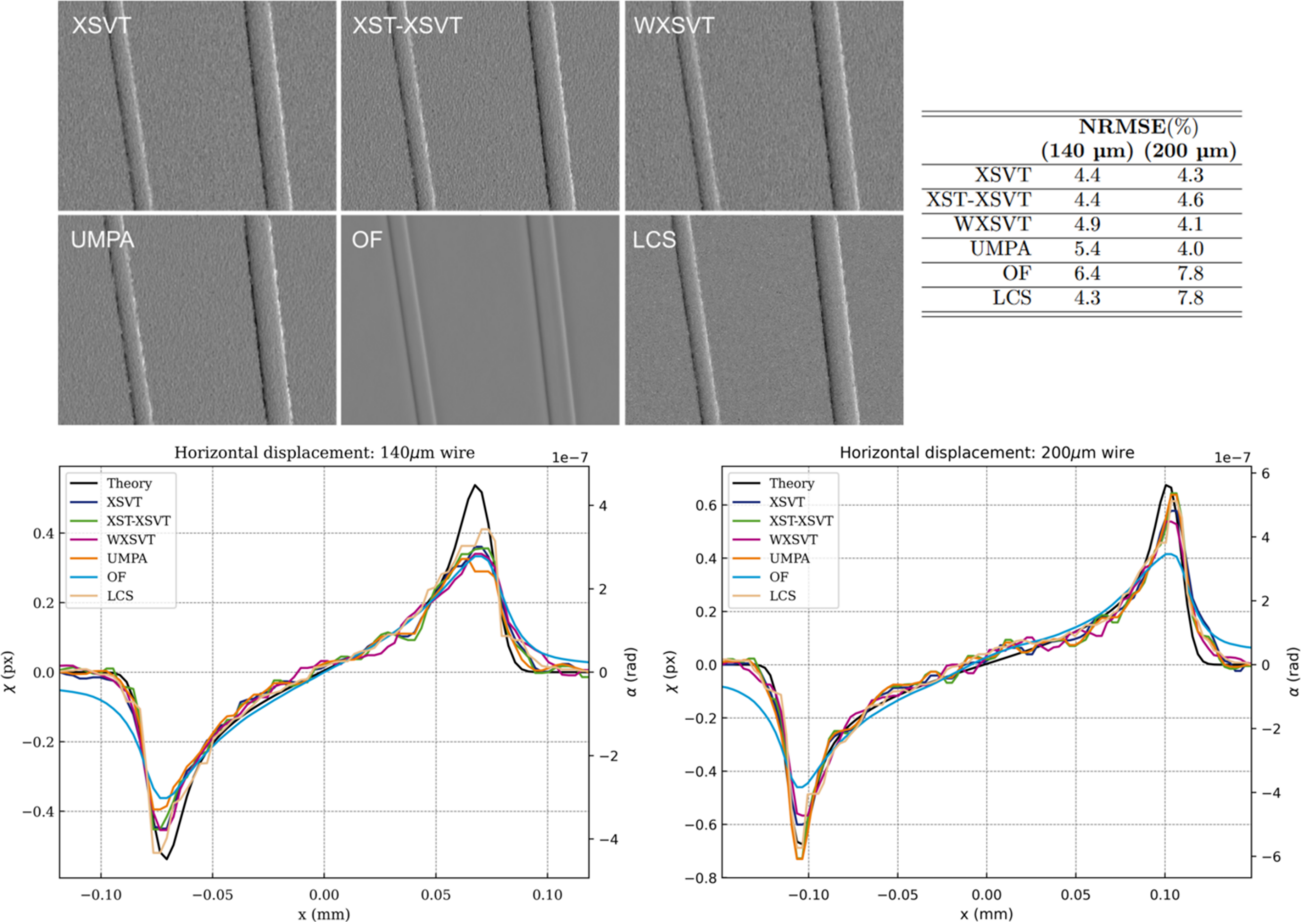
Nylon wires displacement maps retrieved with different algorithms (XSVT, XST–XSVT, WXSVT, UMPA, OF, LCS). Plots of the wires profiles compared with theory. Table of the NRMSE calculated between the experimental profiles and the theoretical one.

This round-robin study was conducted on the nylon wires. The theoretical phase shift induced by a single-material cylindrical sample is known, therefore only the algorithms that retrieve displacement maps were compared: XSVT, XST–XSVT, WXSVT, UMPA, OF and LCS. The phase gradients in this section were obtained by applying the several algorithms to a set of ten reference and ten sample images at different membrane positions. Fig. 2 ▸ shows horizontal displacement for two nylon wires with nominal diameter of 140 µm and 200 µm. Since they are tilted with respect to the vertical axis, the slanted edge method was adopted to obtain a perpendicular profile cut. This method is typically used for the calculation of a super-sampled edge spread function (ESF) or the modulation transfer function (MTF) (Reichenbach *et al.*, 1991 ▸). To reduce noise-induced fluctuations, 200 profiles were averaged per wire in Fig. 2 ▸, which also shows the displacement field in units of pixels and in angular deflection [equation (2)]. A theoretical profile is also shown: it was computed from the gradient of the phase shift induced by a perfect cylindrical nylon wire at the nominal beam energy. The point spread function (PSF) of the detector, here of 2 pixels (Mittone *et al.*, 2017 ▸), was simulated by applying a Gaussian filter to the phase-shift gradient.

In this experiment all algorithms provided a displacement close to the theoretical values, which is confirmed by the calculation of the normalized root-mean-square error (NRMSE) – the NRMSE stays under 5.5% for both wires over a wide choice of algorithms. Possible sources of errors can be difficult to pinpoint, but we list some of them here: regardless of the technique, the edges of the wires are smoothed and this is mainly due to the imaging system lateral resolution (scintillator blurring and imaging system PSF). This is also where the displacement field has a discontinuity; we also bring attention to residual propagation-based edge-enhancement effects, which depend on the Laplacian of the phase-shift; lastly, the non-perfect cylindrical shape of the nylon wires cause difference between observed and calculated values. Optical flow provides the least accurate quantitative information which depends strongly on the high-pass filter applied, despite good qualitative results – for single-material samples, attenuation and phase shift are proportional and related to the thickness of the sample. For this reason, it cannot be excluded that the shape of the profile measured by OF was dominated by the attenuation of the wires.

### 7.2. X-ray lenses and larger pixel displacements

**Figure 3:**
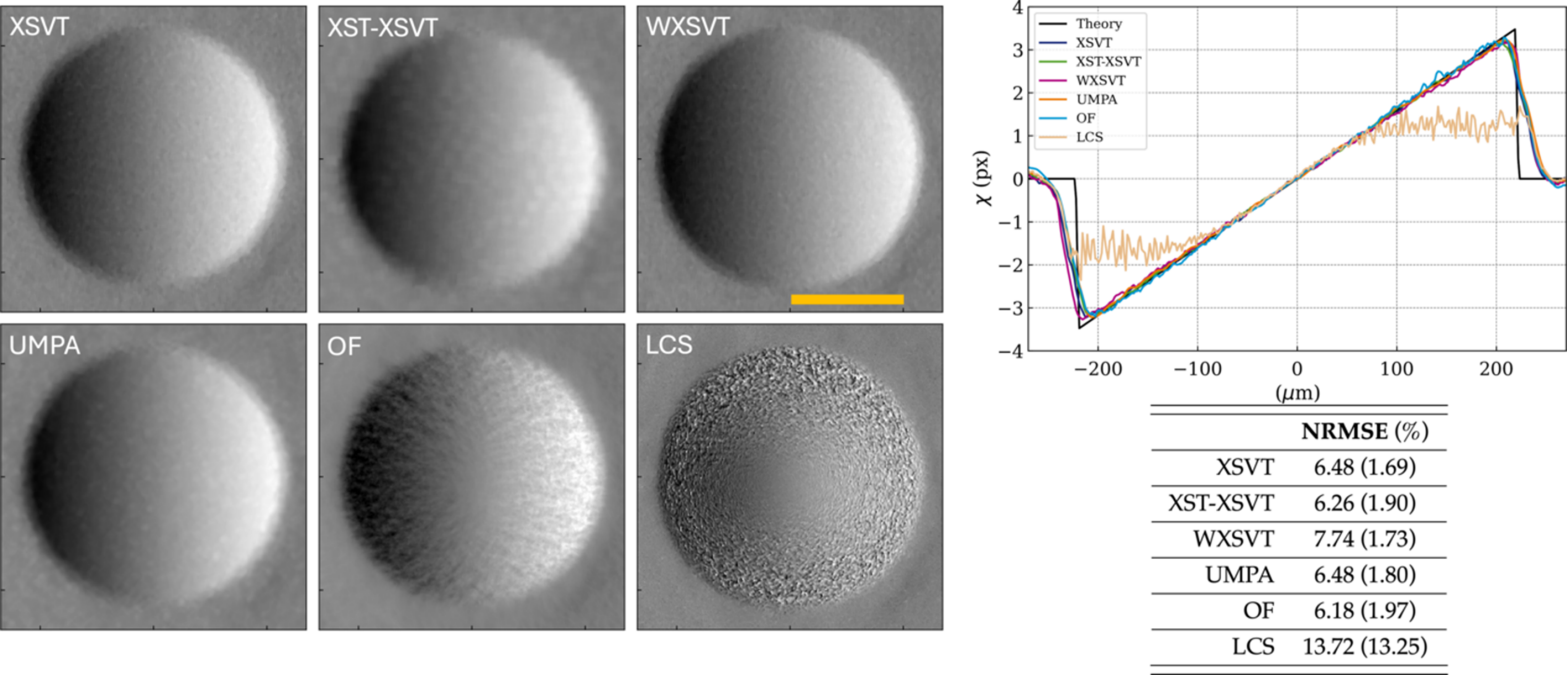
Displacement maps of a 2D beryllium lens with *R* = 50 µm from measurements with ten membrane positions. The NRMSE values are calculated within the limits of the profile cuts above, while values in parentheses are calculated within the lens active area. The horizontal profile cuts were obtained from the centre of the lens. The orange bar represents 200 µm.

A second quantitative study was performed on refractive X-ray lenses with radii varying from 50 µm, 500 µm to 5000 µm – the smaller the radius, the shorter the focal length of the lens and the larger the speckle lateral displacement will be. Under the experimental conditions summarized in Table 1 ▸, their respective focal lengths are 29.36 m, 293.58 m and 2.94 km. X-ray lenses attenuate the incoming beam, but the lenses used in this experiment are made of beryllium and at 20 keV absorption is kept to a minimum. X-ray lenses can be easily modelled and theoretical values for their phase gradients readily obtained (Celestre *et al.*, 2020 ▸; Celestre *et al.*, 2023 ▸). Furthermore, the phase gradients of a 2D parabolic lens is linear in both vertical and horizontal directions within the lens geometric aperture, which makes visual inspection of the displacement graphs in Figs. 3 ▸, 4 ▸ and 5 ▸ straightforward. All these make X-ray lenses good phantoms for SBI and the same treatment dispensed to the nylon wires will be applied to the lenses. Much like in the previous section, the phase gradients presented here were obtained by applying the several algorithms to a set of ten reference and ten sample images at different speckle membrane positions.

Fig. 3 ▸ shows the horizontal phase gradients and the NRMSE values for a 50 µm radius lens. For this experimental configuration, the speckle shift at the edge of the lens is expected to be slightly over 3 pixels. We can see that, with the exception of the LCS algorithm – that stagnates around ±1 pixel, all algorithms follow closely a linear profile and overlap well with the theoretical curve within the lens active area; however, the edges of the lenses are not well represented by the theoretical model, hence the difference between experimental and simulated data reflected by the NRMSE – this mismatch has been documented and discussed into depth in a previous work (Celestre *et al.*, 2023 ▸). The NRMSE vales are calculated within the limits of the profile cuts in Fig. 3 ▸, while the values in parentheses are calculated within the lens active area and hence their lower values (meaning better agreement). A visual inspection of the 2D maps shows that XSVT, WXSVT and UMPA are indistinguishable. The XST–XSVT map is less sharp than the aforementioned methods, but this is already expected due to the method’s intrinsic lower lateral resolution. The results with OF, despite following closely the theoretical profile, present some artefacts/texture that is not observed with the previous methods – this texture is not captured by the NRMSE. The LCS results are not good towards the edge of the lens, where both the displacements and absorption are stronger.

**Figure 4:**
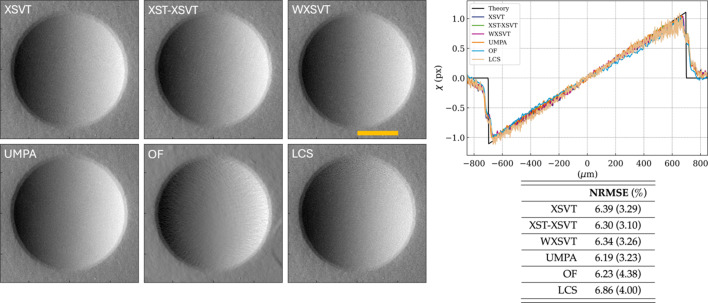
Displacement maps of a 2D beryllium lens with *R* = 500 µm from measurements with ten membrane positions. The NRMSE values are calculated within the limits of the profile cuts above, while values in parentheses are calculated within the lens active area. The horizontal profile cuts were obtained from the centre of the lens. The orange bar represents 500 µm.

Moving to the 500 µm radius lens (Fig. 4 ▸), we see a better agreement between theoretical and experimental values. Here the pixel displacements are confined to ±1 pixel. Much like for the previous lens, XSVT, WXSVT and UMPA are indistinguishable. The XST–XSVT map remains the least sharp method but still presents excellent qualitative and quantitative results. OF still presents some texture near the lens edge, but those are much less prominent. The LCS method behaves much better than for the 50 µm case, but it is still noisy towards the edge of the lens where phase shifts approach the 1 pixel mark. The NRMSE remains between 6.2% and 6.9% for all methods mainly due to the modelling of the lens edge. Explicit tracking methods, however, present slightly lower NRMSE values within the lens active area.

**Figure 5:**
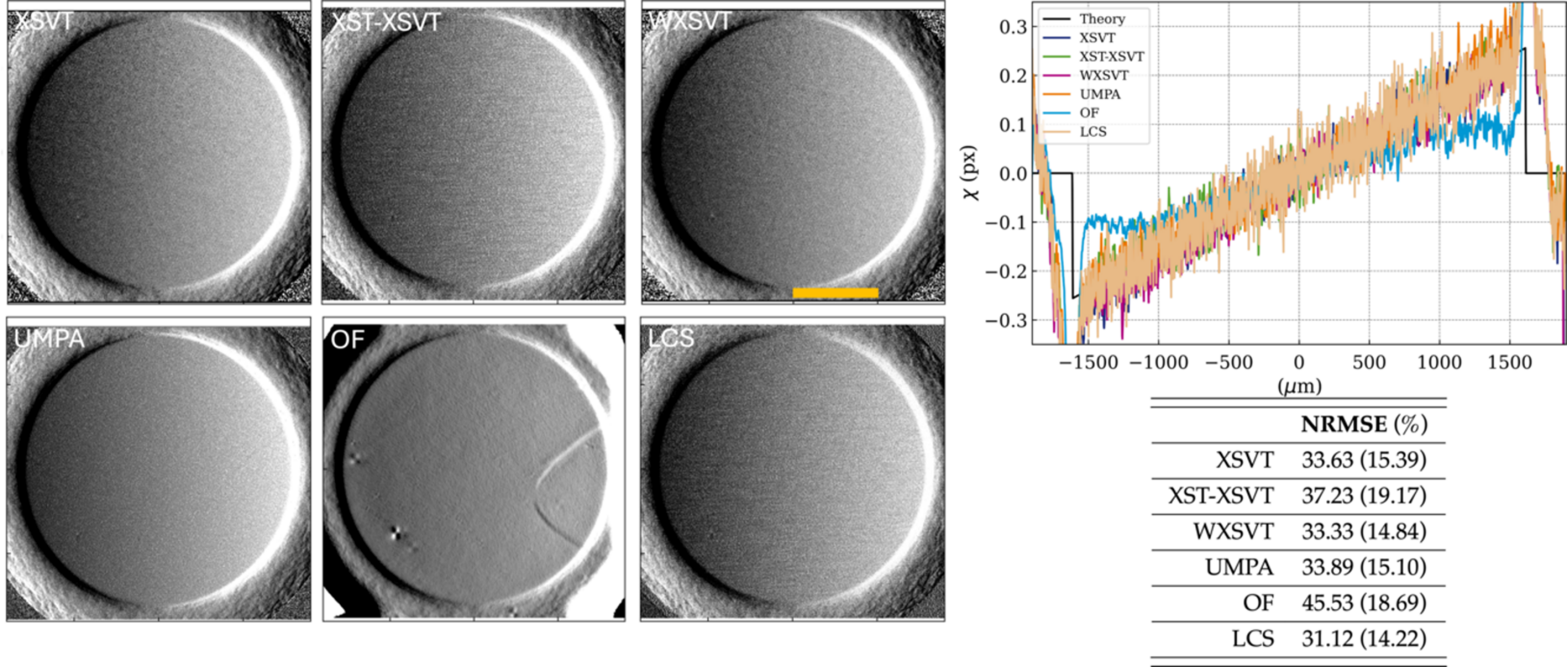
Displacement maps of a 2D beryllium lens with *R* = 5000 µm from measurements with ten membrane positions. The NRMSE values are calculated within the limits of the profile cuts above, while values in parentheses are calculated within the lens active area. The horizontal profile cuts were obtained from the centre of the lens. The orange bar represents 1000 µm.

The third measurement of this series is of a 5000 µm radius lens. This is a very weak phase element and the pixel displacements are below a third of a pixel for the lens geometric aperture – edge effects are rather strong compared with the gradient inside the lens geometric aperture, hence the elevated NRMSE values in Fig. 5 ▸. For this experiment, the explicit tracking methods are rather robust and manage to not only follow the slope of the gradient but also show a slightly lower noise than the LCS method (implicit tracking). The OF algorithm under-performs and does not follow the straight line of the slope as shown by the gradient’s horizontal cuts. The OF image has three noticeable artefacts: two discontinuities on the left side of the lens (singularities/vortexes) and a conic shape on the right side. While the first remains a source of speculation, the latter has also been observed in Fig. 4 ▸ for the same method and most likely comes from the scintillator or the carbon cover of the X-ray imaging system, which shields it from stray light. The same structures are visible in the loss term of LCS, highlighting the different nature of the signal retrieved by OF. The LCS algorithm performs well for this sample, despite higher noise levels in the 2D map. We remind the reader that the noise levels shown here (about one-tenth of a pixel) represent ∼210 nrad of angular sensitivity for this experimental setup.

### 7.3. Mouse knee: qualitative evaluation of a detailed sample under high-coherence illumination

The third study examined a biological sample (mouse knee) using high-coherence illumination. While the previous sections explored the retrieval of quantitative information, this and the following section emphasize image quality. This is because obtaining the theoretical phase shifts produced by this kind of sample is very challenging, if not impossible. Assessing image quality can be very subjective at times. In order to facilitate the qualitative evaluation of the reconstructed phase, we apply a selection of ‘*no-reference*’ image quality ranking methods (IQRMs) commonly used in microscopy – Koho *et al.* (2016 ▸) present a very complete overview of several methods and compare their relative performance; we based our image analysis on their work. To compose our pool of metrics we chose: BRISQUE (blind/referenceless image spatial quality evaluator; Mittal *et al.*, 2012 ▸) and NIQE (natural image quality evaluator; Mittal *et al.*, 2013 ▸) – methods for natural images (MATLAB official implementation); for identifying images with high contrast and continuous spatial features we use the Shannon spatial entropy (sEntropy) and one minus the normalized power spectrum standard deviation (1-fSTD). As pointed out by Koho *et al.* (2016 ▸), the spatial entropy favours images with high contrast, while 1-fSTD should favour non-noisy images. We also add the mean between sEntropy and 1-fSTD (‘mean*’); finally, in order to try to evaluate blur, we chose two frequency-domain metrics: the spectral domain mean (fMean) and standard deviation (fSTD). The implementation of sEntropy, fMean and fSTD was taken from MIPLIB (2020 ▸).

**Figure 6:**
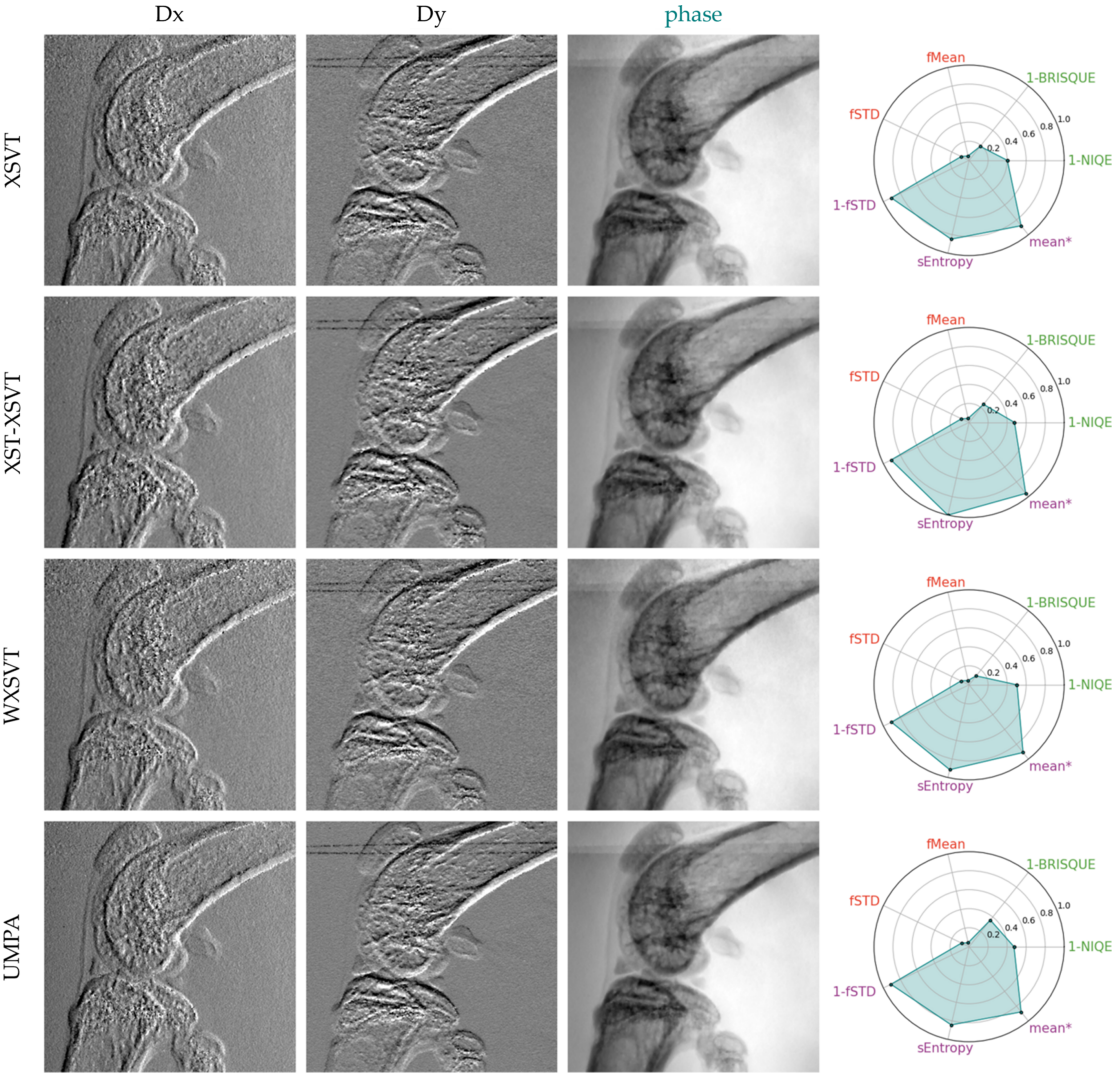
Displacement maps and phase images retrieved from acquisitions at ten membrane positions with various explicit phase retrieval algorithms.

**Figure 8:**
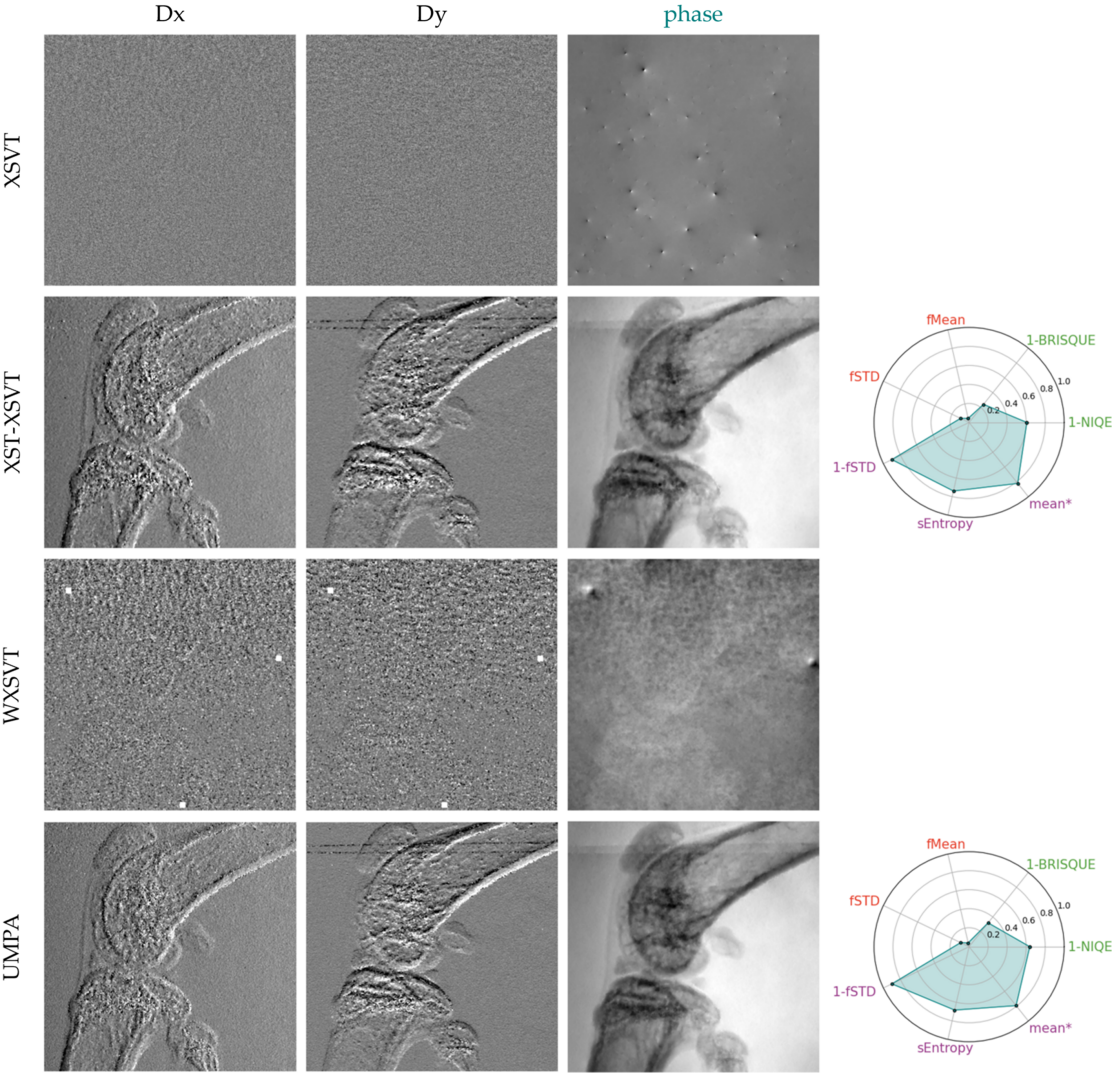
Displacement maps and phase images retrieved from acquisitions at four membrane positions with various explicit phase retrieval algorithms.

We present the phase gradients and reconstructed phases for two data-sets: ten-image-pairs (Figs. 6 ▸ and 7 ▸) and a reduced four-image-pair (Figs. 8 ▸ and 9 ▸) in order to test the robustness of the algorithms to a reduced data-set. The metrics plotted alongside the gradients and reconstructed phases were adjusted by offsetting with the lowest value across both datasets (for each respective metric) and subsequently scaled by dividing by the range of values within each metric. The SMOST algorithm is now included in the pool of tested SBI methods. Unlike the other methods that retrieve phase gradients, SMOST directly obtains the phase. Therefore, Frankot–Chellappa’s integration method (FC), as described in §4, will be applied to obtain the phase for the remainder methods.

Using ten image pairs, all algorithms are able to retrieve phase gradients and the FC integration returns an image that seems to be morphologically correct. Using the aforementioned IQRM, we can say that XSVT, XST–XSVT, WXSVT and UMPA deliver very similar results, with WXSVT having a lower 1-BRISQUE response and UMPA a higher one. OF has the poorest performance on all but 1-STD metric: smooth/blurred images with a reduced dynamic range that makes some details difficult to see. This smoothing can be reduced by tuning the high-pass filter in the Fourier domain but this leads to the appearance of other artefacts. OF assumes a non-absorbing sample, a hypothesis which does not hold with highly absorbing samples such as bones. Since the absorption signal cannot be fully removed from the dataset, the signal retrieved by OF contains the filtered absorption. Isolating only the phase signal of this sample is not possible with this algorithm. The LCS algorithm gives a result that nears the explicit tracking methods, but with a higher fSTD content which translates to a higher granularity in the phase image. LCS has the highest 1-NIQE metric from all images. The SMOST phase image is the one that is subjectively the best in our evaluation: it presents the most details. It has the highest fSTD and fMean when compared with the rest of the ten-image-pair reconstructions. It performs very badly on the 1-NIQE criterion, has very low Shannon entropy but performs well with the 1-BRISQUE. Despite being perceived as the best reconstruction, it shows interference fringes and the image is similar to the one obtained with a simple propagation-based phase imaging setup. This is due to the nature of the retrieved signal that, assuming a single material, combines information from the phase and attenuation. The SMOST equation is similar to the PBI formulation, thus raising doubts about the role of membrane modulation in the image quality.

Now we focus on the reduced dataset. Reconstructions with less experimental data were performed to assess the robustness of the algorithms when minimizing sample exposure to X-rays, thereby reducing the delivered dose – an important consideration for medical imaging applications. Right away, we see that both XSVT and WXSVT fail. This can be explained by the fact that these algorithms track displacements using cross-correlation of 1D arrays with a length of *P*, where *P* is the number of collected images. When *P* is as small as four, the vectors are too short to compute a meaningful cross-correlation. The XST–XSVT and UMPA images become slightly more grainy: fMean is marginally reduced. We also note an increased sEntropy to the images. The 1-NIQE metric is also increased for the smaller set. The LCS is visually worse than the ten-image-pair reconstruction: the background is significantly noisier. The used metrics back up this subjective assertion: fSTD is significantly increased (1-fSTD is decreased) while the entropy plummets. The natural image metrics seem not to be appropriate markers of image quality for this case. The OF is still the least performing from all the algorithms, but it seems to be insensitive to the reduction of image-pairs. SMOST seems to retain its subjective image quality and appears to be unaffected by the data-set reduction, despite having an increase in the fMean and fSTD.

### 7.4. Domestic fly: qualitative evaluation of a detailed sample under low-coherence illumination

**Figure 10:**
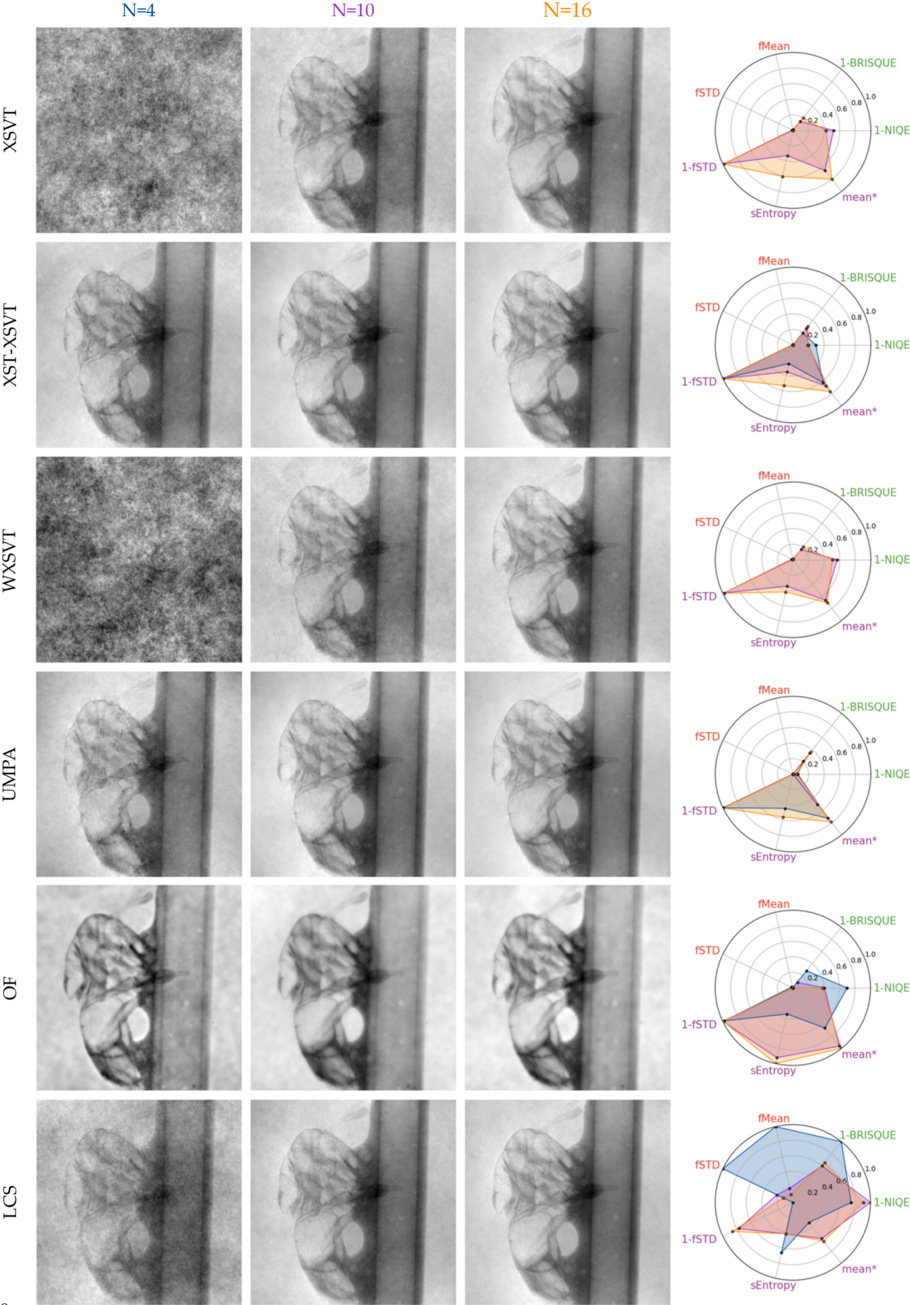
Phase images of a fly retrieved from a conventional set-up set of acquisitions with various available algorithms: XSVT, XST–XSVT, WXSVT, UMPA, OF, LCS from 4, 10 and 16 membrane positions.

Finally, the algorithms were compared in the case of a low-coherence system, by using a laboratory source with polychromatic illumination (a tungsten anode operating at 40 kVp). This experimental setup also has a lower lateral resolution – the detector has a limited pixel size of 48 µm. To compensate for the blurring induced by the detector point spread function, an unsupervised Wiener deconvolution was applied to the acquisitions prior to the phase retrieval. For this comparison, 4, 10 and 16 image pairs were used. The resulting phase images are displayed in Fig. 10 ▸. To facilitate the image quality evaluation, we tried to adopt the same metrics used in the previous section (§7.3); however, a distinctive image quality metric could not be found and the quality assessment remained subjective at times. Basically, for all methods but the OF, it is straightforward to observe an improvement of image quality with increased number of image pairs as they are less grainy – this can be monitored with the background of the image. The metrics fSTD and fMean should be sensitive to it, but the LCS with four image pairs eclipse the results for all the other techniques (metrics are normalized). For the first series (*N* = 4), much like in the previous section, we note that XSVT and WXSVT do not work. UMPA and XST–XSVT give equivalent results with UMPA having a higher entropy, while XST–XSVT has a higher fMean and fSTD but the perceived difference in the images is marginal. For an increased number of images – be it 10 or 16 – XSVT, XST–XSVT, UMPA and LCS appear to give equivalent results, despite XST–XSVT, UMPA having a cleaner background. Confirming what was observed in §7.3, OF seems to be invariant to the number of images used in the reconstruction – while there is a difference between *N* = 4 and the rest of the OF reconstructions, *N* = 10 and *N* = 16 are very similar. It is clear upon visual inspection that those images appear to be less sharp than the other methods, which is related to how the several reconstructions are merged together (Appendix *A*[App appa]). It is difficult, however to rank them amongst each other. Using this same metric, we can see that OF presents the least sharp of the image reconstructions. But in this case, the blur is balanced with an edge enhancement effect due to the Fourier frequency filter. It appears to be the least noisy result; however, the nature of the signal retrieved might be mainly due to filtered attenuation as previously discussed.

## 8. Conclusions and perspectives

Table 2 ▸ summarize the results obtained with the different algorithms and Appendix *A*[App appa] summarizes all the relevant parameters for phase retrieval used in this work. These methods present different advantages and drawbacks which are summarized in §2.7 and §3.5. If searching for high displacement quantitative measures, the XSVT, XST–XSVT, WXSVT and UMPA are the best options. The WXSVT has the advantage of a lower computational cost if fewer wavelet coefficients are used. For small displacements, the LCS gives quantitative results and might be the fastest computation option. For synchrotron high-resolution imaging, XSVT, XST–XSVT, WXSVT, LCS and UMPA give similar results when a large number of membrane positions are acquired. With smaller numbers of points, XSVT and WXSVT are no longer good candidates. For conventional micro-focus source imaging of complex samples, the LCS, UMPA and XST–XSVT appear to give the best results that are quite similar to each other. Once again, for faster computation, the LCS is the best solution. OF and SMOST give interesting results when looking at complex samples but the nature of the extracted signal remains an issue. Further tests should be done to completely identify and characterize it. For example, the same SBI experiment could be performed with and without the modulator, to study the role of the membrane for signal extraction. A measurement without membrane would moreover provide a reliable measurement of the absorption, which could be decoupled from the phase signal.

## Figures and Tables

**Figure 7:**
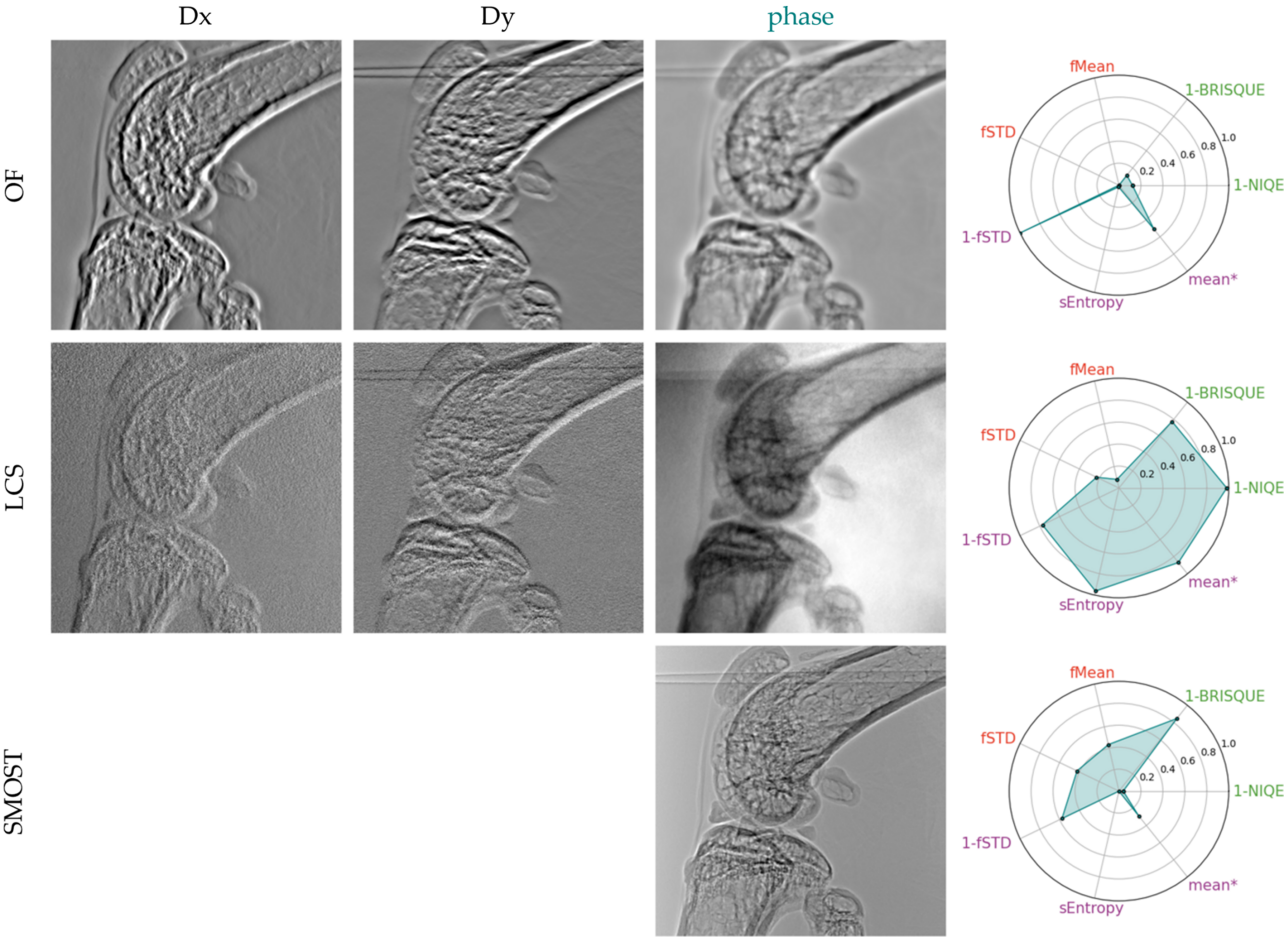
Displacement maps and phase images retrieved from acquisitions at ten membrane positions with various implicit phase retrieval algorithms.

**Figure 9:**
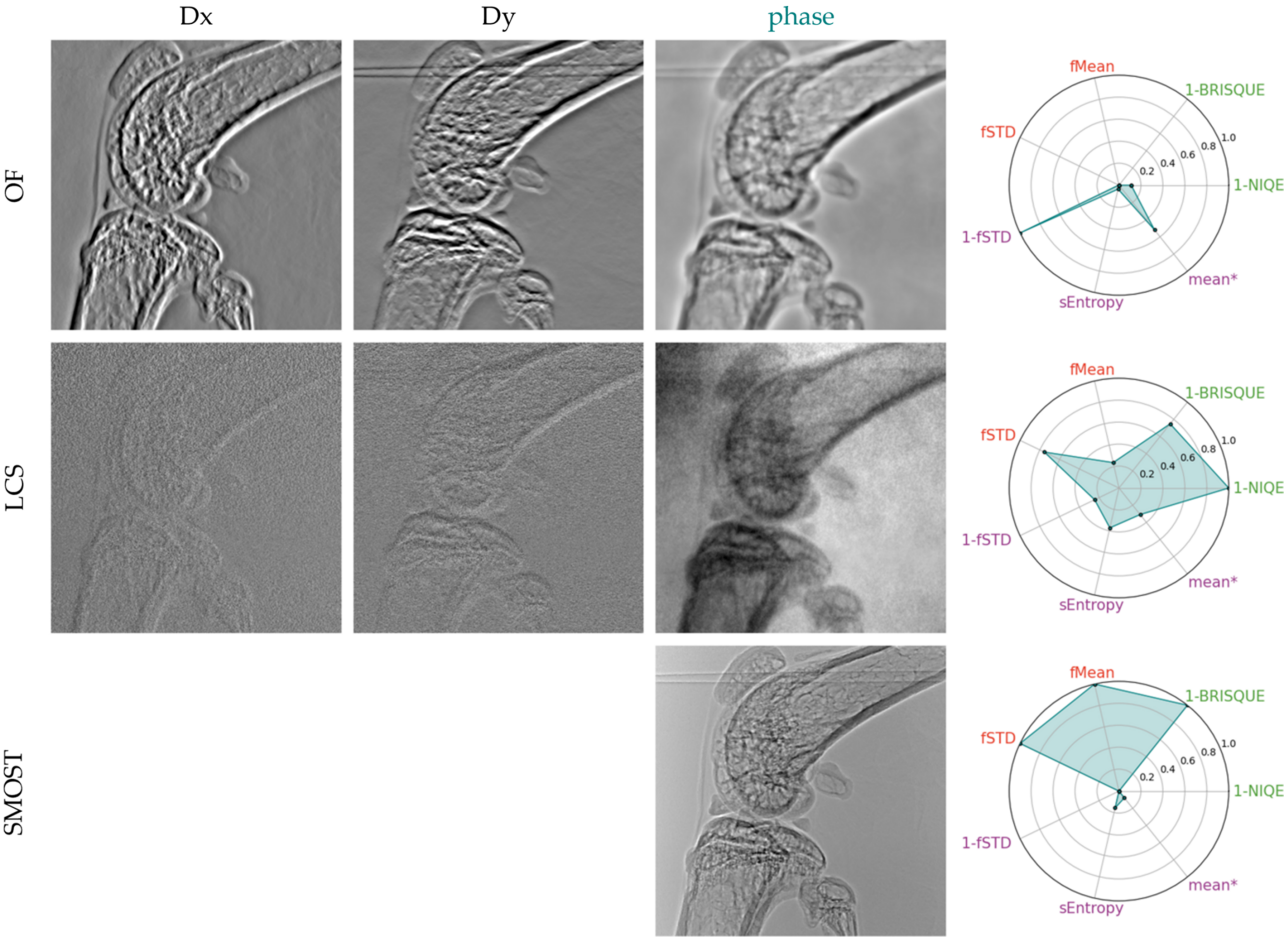
Displacement maps and phase images retrieved from acquisitions at ten membrane positions with various implicit phase retrieval algorithms.

**Figure 11:**
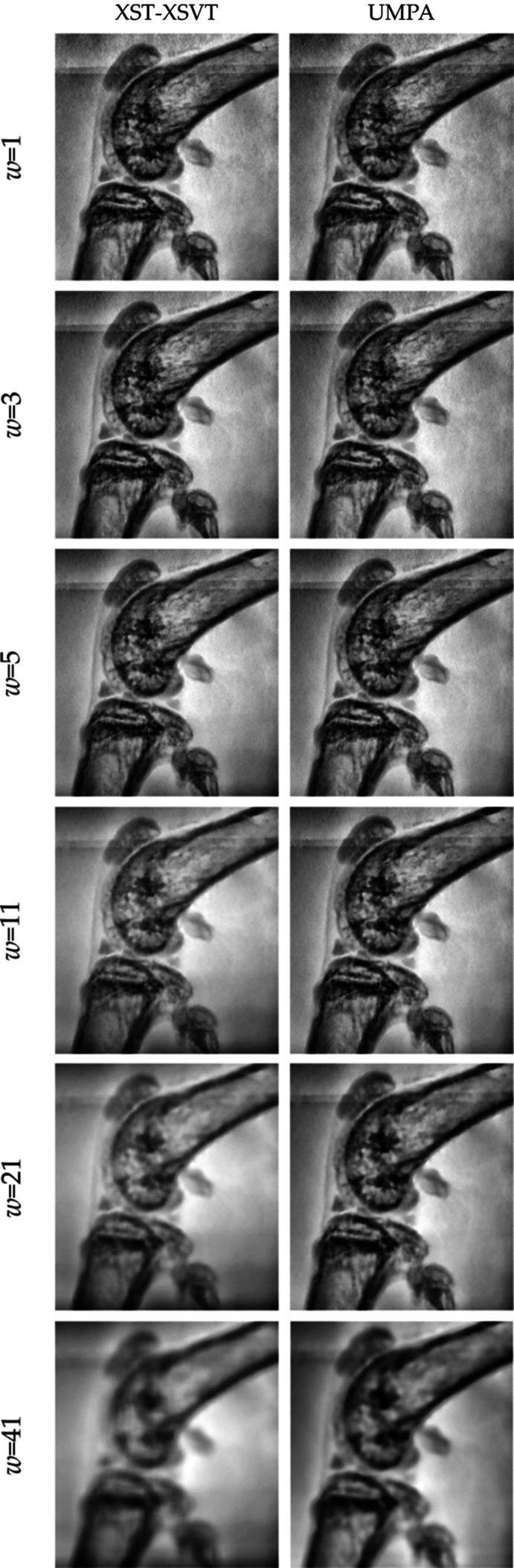
Reconstructed phase image of the mouse knee from §7.3 for ten sample/reference image pairs for increasing *w* with 2*m* + 1 = 2*n* + 1.

**Figure 12:**
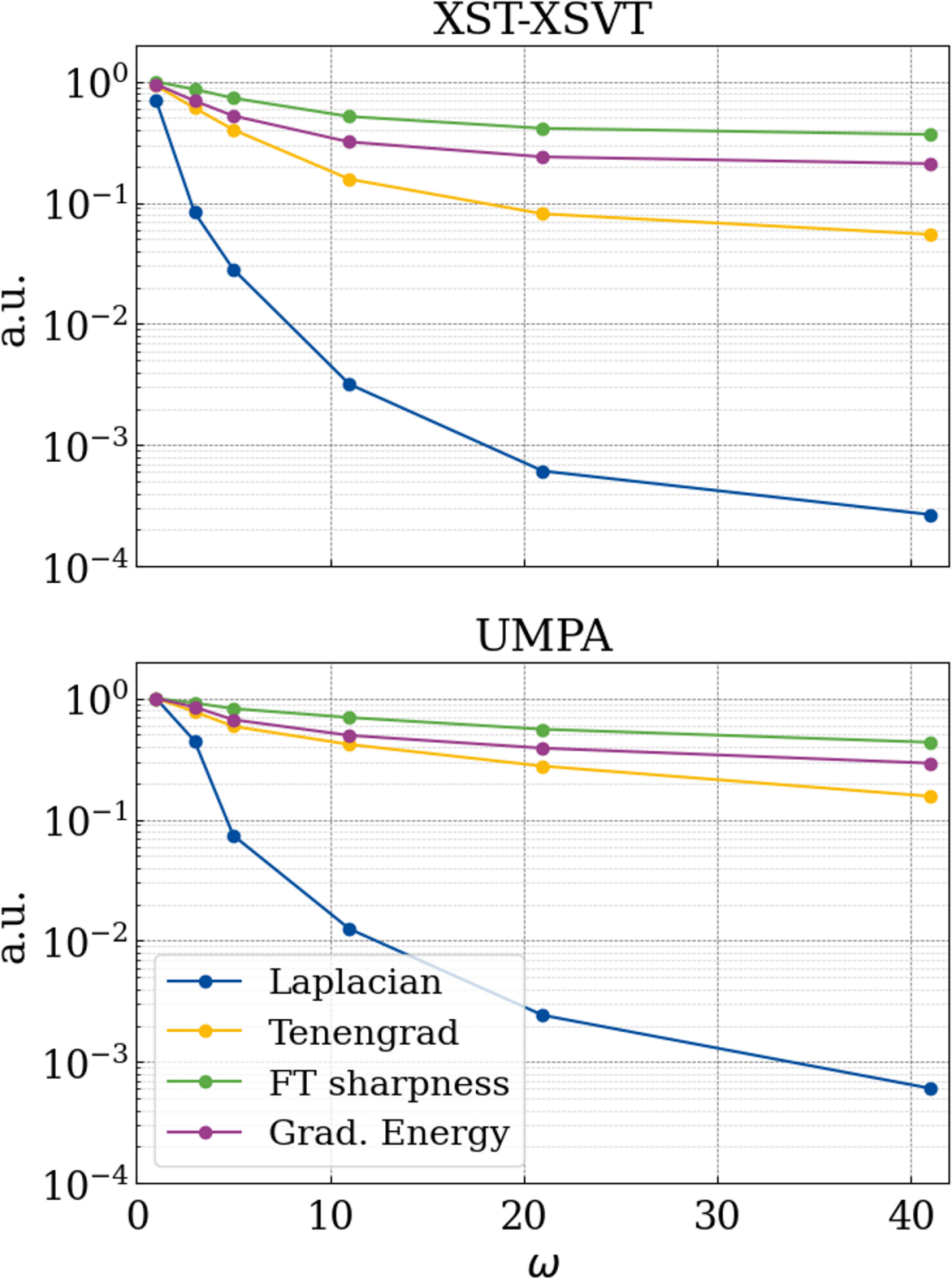
Metrics for assessing the sharpness of the reconstructed phase images in Fig. 11 ▸.

**Table:** 

Sample	Source	Beam energy (keV)	Propagation distance (m)
Nylon wires (Ø140 µm, Ø200 µm)	ID17	52.0	3.600
X-ray lenses (50 µm, 500 µm, 5000 µm)	BM05	20.0	0.750
Mouse knee	ID17	52.0	11.000
Fly on straw	Easytom XL-RX	W anode at 40.0 kVp	0.345

**Table:** 

Sample	Beam modulator	Pixel size (µm)	Detector	Section (§)
Nylon wires (Ø140 µm, Ø200 µm)	Sandpaper	3.0	pco.edge	7.1
X-ray lenses (50 µm, 500 µm, 5000 µm)	Filter membrane (pore size ∼1.2 µm)	1.6	pco.edge	7.2
Mouse knee	sandpaper	6.1	pco.edge	7.3
Fly on straw	TiC powder (grain size ∼100 µm)	48.0	Varian flat panel	7.4

**Table:** 

	Explicit tracking			
	XSVT	XST–XSVT	WXSVT	UMPA
Section (§)	2.2	2.3	2.4	2.6
Quantitative	Yes	Yes	Yes	Yes
Sensitivity to small pixel displacements	+	+	+	+
Sensitivity to large pixel displacements	+	+	+	+
Robust to decrease in membrane positions	−	+	−	+
Low coherence	+	+	+	+
Computational cost	High	Very high	High	Very high

**Table:** 

	Implicit tracking		
	OF	SMOST	LCS
Section (§)	3.1	3.2	3.3
Quantitative	No	No due to filtering in the Fourier domain	Yes for sub-pixel displacements
Sensitivity to small pixel displacements	/	/	++
Sensitivity to large pixel displacements	/	/	−
Robust to decrease in membrane positions	++	++	± (>3)
Low coherence	+	/	+
Computational cost	Very low	Very low	Low

## Footnotes

1 See also Hu *et al.* (2024 ▸) for other numerical recipes.

## Appendix A Phase retrieval parameters

### A1. XSVT

The XSVT processing of experimental data was consistent across all sample types and closely followed the method described by Berujon *et al.* (2020*b* ▸). Each speckle vector (1D) was searched within a window of size *W* = (2*M* + 1) × (2*N* + 1) with *M* = *N* = 10. For XSVT, as long as the window is large enough to contain the matched signal, reducing the window size only speeds up the calculations. This was not a concern at the time, as the computations were performed on the ESRF SLURM cluster using 40 cores in parallel, taking less than a minute for a 2159 × 2559 pixel image using ten sample/reference pairs. We tracked local normalized cross-correlation values, and if a pixel’s correlation fell below a threshold – indicating a poor match – we locally increased the search window to improve the signal match. Sub-pixel tracking of the maxima is done using equations (7) and (8) from Qiao *et al.* (2020*a* ▸). The phase gradients then underwent post-processing, where a 5 × 5 median filter was applied locally only to outlier values to avoid altering the unaffected parts of the image.

### A2. XST–XSVT

The XST–XSVT algorithm is more computationally demanding than its XST counterpart. It was implemented outside the ESRF, therefore the ESRF-SLURM cluster was not utilized for the analysis. The size of the window *w* was kept as small as possible, *i.e.* 3 × 3 pixels. The search interval *W* was selected based on the maximum lateral displacement expected for each sample, to minimize the computational time. *W* was set to 5 × 5 for the 50 µm Be lense, 3 × 3 for the 500 µm and 5000 µm lenses and the wires, and reduced to 2 × 2 only for the biomedical samples: the knee and the fly.

### A3. WXSVT

The data processing using WXSVT was identical to XSVT in terms of search window sizes, sub-pixel tracking and post-processing of outliers. To achieve the best results, we retained all wavelet coefficients, prioritizing accuracy over computation time. Despite this, calculations for ten sample/reference pairs (2159 × 2559 pixels) still took less than a minute using the ESRF SLURM cluster with 40 cores in parallel.

### A4. UMPA

The analysis with the UMPA algorithm was identical to XST–XSVT.

### A5. OF

The OF algorithm was run on every sample/reference pair and *D*_⊥_(*x*, *y*) was computed as the median of the resulting displacement maps. The threshold of the Gaussian-shaped high-pass filter strongly affects the resulting *D*_⊥_(*x*, *y*) and had to be manually selected for each dataset to optimize the image quality.

### A6. SMOST

Similarly to OF, SMOST can run on a single sample/reference pair. When more image pairs are used, the median of the displacement maps was considered as the resulting *D*_⊥_(*x*, *y*).

### A7. LCS

The LCS algorithm does not require the manual selection of any parameters. The linear system in equation (20) was solved via QR (Golub & Van Loan, 2013 ▸) decomposition.

## Appendix B Effects of the window size on the lateral resolution of the XST–XSVT and UMPA methods

In the sections on the XST–XSVT (Berujon & Ziegler, 2016 ▸) and UMPA (Zanette *et al.*, 2014 ▸; Zdora *et al.*, 2017 ▸) methods (§2.3 and §2.6) we briefly mentioned the reduction in lateral resolution of the reconstructed phase (or gradients) with increasing window size but did not explore it further in §7. This limitation has already been thoroughly addressed in the original publications and examined in detail by Tian *et al.* (2020*b* ▸). Figs. 11 ▸ and 12 ▸ show the decrease in lateral resolution for the XST–XSVT and UMPA methods for increasing window size. In order to facilitate the visual comparison, the plots in Fig. 11 ▸ are shown using the contrast limited adaptive histogram equalization (CLAHE) normalization from OpenCV (OpenCV, 2024 ▸). We notice that the the XST–XSVT has a worse lateral resolution than the UMPA method for the same *w* (in this example 2*m* + 1 = 2*n* + 1) – see Fig. 12 ▸. We used the Laplacian variance (LAP4), the Tenengrad or gradient magnitude (GRA6) and gradient energy (GRA2) from Pertuz *et al.* (2013 ▸) and the high frequency content of the Fourier transform of the image (‘FT sharpness’). The accompanying increase in angular sensitivity is easy to understand given the larger size of the motif being correlated in the search window and the accuracy of sub-pixel maxima tracking methods (Fisher & Naidu, 1996 ▸).

## References

[bb1] Alloo, S. J., Morgan, K. S., Paganin, D. M. & Pavlov, K. M. (2023). *Sci. Rep.* **13**, 5424. 10.1038/s41598-023-31574-z PMC10070351 37012270

[bb2] Alloo, S. J., Paganin, D. M., Morgan, K. S., Kitchen, M. J., Stevenson, A. W., Mayo, S. C., Li, H. T., Kennedy, B. M., Maksimenko, A., Bowden, J. C. & Pavlov, K. M. (2022). *J. Med. Imag.* **9**, 031502. 10.1117/1.JMI.9.3.031502 PMC8820385 35155717

[bb3] Arnison, M. R., Larkin, K. G., Sheppard, C. J., Smith, N. I. & Cogswell, C. J. (2004). *J. Microsc.* **214**, 7–12. 10.1111/j.0022-2720.2004.01293.x 15049862

[bb4] Berujon, S., Cojocaru, R., Piault, P., Celestre, R., Roth, T., Barrett, R. & Ziegler, E. (2020*a*). *J. Synchrotron Rad.* **27**, 293–304. 10.1107/S1600577520000508 32153268

[bb5] Berujon, S., Cojocaru, R., Piault, P., Celestre, R., Roth, T., Barrett, R. & Ziegler, E. (2020*b*). *J. Synchrotron Rad.* **27**, 284–292. 10.1107/S1600577520000491 32153267

[bb6] Berujon, S., Wang, H. & Sawhney, K. (2012). *Phys. Rev. A*, **86**, 063813.

[bb7] Berujon, S. & Ziegler, E. (2015). *Phys. Rev. A*, **92**, 013837.

[bb8] Berujon, S. & Ziegler, E. (2016). *Phys. Rev. Appl.* **5**, 044014.

[bb9] Bérujon, S., Ziegler, E., Cerbino, R. & Peverini, L. (2012). *Phys. Rev. Lett.* **108**, 158102. 10.1103/PhysRevLett.108.158102 22587288

[bb10] Berujon, S., Ziegler, E. & Cloetens, P. (2015). *J. Synchrotron Rad.* **22**, 886–894. 10.1107/S1600577515005433 PMC4787027 26134791

[bb11] Bon, P., Monneret, S. & Wattellier, B. (2012). *Appl. Opt.* **51**, 5698–5704. 10.1364/AO.51.005698 22885583

[bb12] Bonse, U. & Hart, M. (1965). *Appl. Phys. Lett.* **6**, 155–156.

[bb13] Brunetti, A., Sanchez del Rio, M., Golosio, B., Simionovici, A. & Somogyi, A. (2004). *At. Spectrosc.* **59**, 1725–1731.

[bb14] Celestre, R., Chubar, O., Roth, T., Sanchez del Rio, M. & Barrett, R. (2020). *Proc. SPIE*, **11493**, 114930J.

[bb15] Celestre, R., Roth, T., Detlefs, C., Qi, P., Cammarata, M., Sanchez del Rio, M. & Barrett, R. (2023). *Opt. Express*, **31**, 7617–7631. 10.1364/OE.481678 36859890

[bb16] Cerbino, R., Peverini, L., Potenza, M. A. C., Robert, A., Bösecke, P. & Giglio, M. (2008). *Nat. Phys.* **4**, 238–243.

[bb17] Cloetens, P., Barrett, R., Baruchel, J., Guigay, J.-P. & Schlenker, M. (1996). *J. Phys. D Appl. Phys.* **29**, 133–146.

[bb18] Cunningham, J. A., Rahman, I. A., Lautenschlager, S., Rayfield, E. J. & Donoghue, P. C. (2014). *Trends Ecol. Evol.* **29**, 347–357. 10.1016/j.tree.2014.04.004 24821516

[bb777] David, C., Nöhammer, B., Solak, H. H. & Ziegler, E. (2002). *Appl. Phys. Lett.* **81**, 3287–3289.

[bb19] De Marco, F., Savatović, S., Smith, R., Di Trapani, V., Margini, M., Lautizi, G. & Thibault, P. (2023). *Opt. Express*, **31**, 635–650. 10.1364/OE.474794 36606998

[bb20] Dos Santos Rolo, T., Reich, S., Karpov, D., Gasilov, S., Kunka, D., Fohtung, E., Baumbach, T. & Plech, A. (2018). *Appl. Sci.* **8**, 737.

[bb21] Endrizzi, M. (2018). *Nucl. Instrum. Methods Phys. Res. A*, **878**, 88–98.

[bb22] Ettl, S., Kaminski, J., Knauer, M. C. & Häusler, G. (2008). *Appl. Opt.* **47**, 2091–2097. 10.1364/ao.47.002091 18425183

[bb23] Fisher, R. B. & Naidu, D. K. (1996). *A Comparison of Algorithms for Subpixel Peak Detection*, pp. 385–404. Berlin, Heidelberg: Springer.

[bb24] Förster, E., Goetz, K. & Zaumseil, P. (1980). *Cryst. Res. Technol.* **15**, 937–945.

[bb25] Frankot, R. T. & Chellappa, R. (1988). *IEEE Trans. Pattern Anal. Mach. Intell.* **10**, 439–451.

[bb26] Geloni, G., Saldin, E., Schneidmiller, E. & Yurkov, M. (2008). *Nucl. Instrum. Methods Phys. Res. A*, **588**, 463–493.

[bb779] Golub, G. H. & Van Loan, C. F. (2013). *Matrix Computations*, 4th ed., ch. 5.2, p. 246. John Hopkins University Press.

[bb27] Gureyev, T. E., Kozlov, A., Paganin, D. M., Nesterets, Y. I., De Hoog, F. & Quiney, H. M. (2017). *J. Opt. Soc. Am. A*, **34**, 1577–1584. 10.1364/JOSAA.34.001577 29036160

[bb28] Gureyev, T. E., Nesterets, Y. I. & Paganin, D. M. (2015). *Phys. Rev. A*, **92**, 053860.

[bb29] Gureyev, T. E., Nesterets, Y. I., Paganin, D. M. & Wilkins, S. W. (2006). *J. Opt. Soc. Am. A*, **23**, 34–42. 10.1364/josaa.23.000034 16478058

[bb30] Gureyev, T. E., Paganin, D. M. & Quiney, H. M. (2024). *J. Synchrotron Rad.* **31**, 896–909. 10.1107/S1600577524003886 PMC11226163 38843003

[bb31] Hall, C. (2021). *Joint IAEA-ANSTO Workshop on Nuclear and Isotopic Techniques for Cultural Heritage*, 6–9 December 2021, North Wollongong, NSW, Australia. Vienna: IAEA.

[bb32] Henke, B., Gullikson, E. & Davis, J. (1993). *At. Data Nucl. Data Tables*, **54**, 181–342.

[bb33] Hu, L., Wang, H. & Sawhney, K. (2024). *J. Synchrotron Rad.* **31**, 1037–1042. 10.1107/S1600577524005861 PMC11371044 39078691

[bb34] Huang, L., Idir, M., Zuo, C., Kaznatcheev, K., Zhou, L. & Asundi, A. (2015). *Opt. Lasers Eng.* **64**, 1–11.

[bb35] Hubbell, J. H., Veigele, W. J., Briggs, E. A., Brown, R. T., Cromer, D. T. & Howerton, R. J. (1975). *J. Phys. Chem. Ref. Data*, **4**, 471–538.

[bb36] Kashyap, Y., Wang, H. & Sawhney, K. (2016). *Rev. Sci. Instrum.* **87**, 052001. 10.1063/1.4949004 27250381

[bb37] Koho, S., Fazeli, E., Eriksson, J. E. & Hänninen, P. E. (2016). *Sci. Rep.* **6**, 28962. 10.1038/srep28962 PMC4929473 27364703

[bb38] Kottler, C., David, C., Pfeiffer, F. & Bunk, O. (2007). *Opt. Express*, **15**, 1175–1181. 10.1364/oe.15.001175 19532346

[bb40] Labriet, H., Berujon, S. & Brun, E. (2022). *X-ray imaging device and associated imaging method.* Patent: US 20220273254 A1. Filed: 30 June 2020; status: pending (https://patents.google.com/patent/US20220273254A1/en).

[bb39] La Rochefoucauld, O. de, Dovillaire, G., Harms, F., Idir, M., Huang, L., Levecq, X., Piponnier, M. & Zeitoun, P. (2021). *Sensors*, **21**, 874. 10.3390/s21030874 PMC7865934 33525501

[bb41] Lengeler, B., Schroer, C. G., Kuhlmann, M., Benner, B., Günzler, T. F., Kurapova, O., Somogyi, A., Snigirev, A. & Snigireva, I. (2004). *AIP Conf. Proc.* **705**, 748–751.

[bb42] Mayo, S. C. & Sexton, B. (2004). *Opt. Lett.* **29**, 866. 10.1364/ol.29.000866 15119404

[bb43] Mercère, P., Zeitoun, P., Idir, M., Le Pape, S., Douillet, D., Levecq, X., Dovillaire, G., Bucourt, S., Goldberg, K. A., Naulleau, P. P. & Rekawa, S. (2003). *Opt. Lett.* **28**, 1534. 10.1364/ol.28.001534 12956370

[bb44] Mikhaylov, A., Reich, S., Zakharova, M., Vlnieska, V., Laptev, R., Plech, A. & Kunka, D. (2020). *J. Synchrotron Rad.* **27**, 788–795. 10.1107/S1600577520002830 PMC7206553 32381782

[bb45] MIPLIB (2020). *MIPLIB*, https://github.com/sakoho81/miplib/ [accessed on 5 September 2024].

[bb46] Mittal, A., Moorthy, A. K. & Bovik, A. C. (2012). *IEEE Trans. Image Process.* **21**, 4695–4708. 10.1109/TIP.2012.2214050 22910118

[bb47] Mittal, A., Soundararajan, R. & Bovik, A. C. (2013). *IEEE Signal Process. Lett.* **20**, 209–212.

[bb48] Mittone, A., Manakov, I., Broche, L., Jarnias, C., Coan, P. & Bravin, A. (2017). *J. Synchrotron Rad.* **24**, 1226–1236. 10.1107/S160057751701222X 29091066

[bb49] Momose, A. (2020). *Phys. Med.* **79**, 93–102. 10.1016/j.ejmp.2020.11.003 33212423

[bb50] Morgan, K. S., Paganin, D. M. & Siu, K. K. (2011). *Opt. Express*, **19**, 19781–19789. 10.1364/OE.19.019781 21996920

[bb51] Morgan, K. S., Paganin, D. M. & Siu, K. K. (2012). *Appl. Phys. Lett.* **100**, 124102.

[bb52] OpenCV (2024). *Histograms – 2: Histogram Equalization*, https://tinyurl.com/opencv-clahe [accessed on 16 September 2024].

[bb53] Paganin, D. (2006). *Coherent X-ray Optics*, 1st ed. Oxford University Press.

[bb54] Paganin, D., Mayo, S. C., Gureyev, T. E., Miller, P. R. & Wilkins, S. W. (2002). *J. Microsc.* **206**, 33–40. 10.1046/j.1365-2818.2002.01010.x 12000561

[bb55] Paganin, D. M., Labriet, H., Brun, E. & Berujon, S. (2018). *Phys. Rev. A*, **98**, 053813.

[bb56] Paganin, D. M. & Morgan, K. S. (2019). *Sci. Rep.* **9**, 17537. 10.1038/s41598-019-52284-5 PMC6879762 31772186

[bb57] Pavlov, K. M., Li, H., Paganin, D. M., Berujon, S., Rougé-Labriet, H. & Brun, E. (2020*a*). *Phys. Rev. Appl.* **13**, 054023.

[bb59] Pavlov, K. M., Paganin, D. M., Morgan, K. S., Li, H. T., Berujon, S., Quénot, L. & Brun, E. (2021). *Phys. Rev. A*, **104**, 053505.

[bb58] Pavlov, K. M., Paganin, D. M., Li, H. (T.), Berujon, S., Rougé-Labriet, H. & Brun, E. (2020*b*). *J. Opt.* **22**, 125604.

[bb60] Pertuz, S., Puig, D. & Garcia, M. A. (2013). *Pattern Recognit.* **46**, 1415–1432.

[bb61] Ponchut, C., Tartoni, N. & Pennicard, D. (2021). *Radiat. Meas.* **140**, 106459.

[bb62] POPCORN (2021). *Popcorn*, https://github.com/doctoremmetbrown/popcorn/ [accessed on 15 June 2023].

[bb63] Qiao, Z., Shi, X. & Assoufid, L. (2020*b*). In *Proc. SPIE*, **11492**, 114920O.

[bb64] Qiao, Z., Shi, X., Celestre, R. & Assoufid, L. (2020*a*). *Opt. Express*, **28**, 33053–33067. 10.1364/OE.404606 33114975

[bb65] Qiao, Z., Shi, X., Wojcik, M. & Assoufid, L. (2021). *J. Imaging*, **7**, 249. 10.3390/jimaging7120249 PMC8705295 34940716

[bb66] Qiao, Z., Shi, X., Yao, Y., Wojcik, M. J., Rebuffi, L., Cherukara, M. J. & Assoufid, L. (2022). *Optica*, **9**, 391–398.

[bb67] Quenot, L., Bohic, S. & Brun, E. (2022). *Appl. Sci.* **12**, 9539.

[bb68] Quénot, L., Broche, L., Tavakoli, C., Bohic, S. & Brun, E. (2021*b*). *Proc. SPIE*, pp. **11595**, 115954M.

[bb69] Quénot, L., Brun, E., Létang, J.-M. & Langer, M. (2021*a*). *Phys. Med. Biol.* **66**, 175027. 10.1088/1361-6560/ac1f38 34412046

[bb70] Quénot, L., Rougé-Labriet, H., Bohic, S., Berujon, S. & Brun, E. (2021*c*). *Optica*, **8**, 1412–1415. 10.1088/1361-6560/ab87f7 32268312

[bb82] Refactored UMPA (2022). *UMPA*, https://github.com/optimato/umpa/ [accessed on 15 June 2023].

[bb71] Reichenbach, S. E. (1991). *Opt. Eng.* **30**, 170–177.

[bb72] Rouge-Labriet, H., Quenot, L., Bohic, S., Fayard, B., Paganin, D. M., Brun, E. & Berujon, S. (2021*a*). *Phys. Med. Biol.* **66**, 065005. 10.1088/1361-6560/ab87f7 32268312

[bb73] Rouge-Labriet, H., Quenot, L., Bohic, S., Fayard, B., Paganin, D. M., Brun, E. & Berujon, S. (2021*b*). *Phys. Med. Biol.* **66**, 065005. 10.1088/1361-6560/ab87f7 32268312

[bb74] Seaberg, M., Cojocaru, R., Berujon, S., Ziegler, E., Jaggi, A., Krempasky, J., Seiboth, F., Aquila, A., Liu, Y., Sakdinawat, A., Lee, H. J., Flechsig, U., Patthey, L., Koch, F., Seniutinas, G., David, C., Zhu, D., Mikeš, L., Makita, M., Koyama, T., Mancuso, A. P., Chapman, H. N. & Vagovič, P. (2019). *J. Synchrotron Rad.* **26**, 1115–1126. 10.1107/S1600577519005721 PMC6613120 31274435

[bb75] Shi, X., Qiao, Z., Wojcik, M. J. & Assoufid, L. (2023). *Coded-mask-based X-ray phase-contrast and dark-field imaging.* Patent: US 11701077 B2. Filed: 25 February 2021; granted: 18 July 2023 (https://patents.google.com/patent/US11701077B2/en).

[bb76] Siano, M., Paroli, B. & Potenza, M. A. C. (2021). *Adv. Phys. X*, **6**, 1891001.

[bb77] Snigirev, A., Snigireva, I., Kohn, V., Kuznetsov, S. & Schelokov, I. (1995). *Rev. Sci. Instrum.* **66**, 5486–5492.

[bb78] Sun, C. (2002). *Image Vis. Comput.* **20**, 981–991.

[bb79] Tian, N., Jiang, H., Li, A., Liang, D., Yan, S. & Zhang, Z. (2020*a*). *J. Synchrotron Rad.* **27**, 146–157. 10.1107/S1600577519015200 31868747

[bb80] Tian, N., Jiang, H., Li, A., Liang, D. & Yu, F. (2020*b*). *Sci. Rep.* **10**, 14238. 10.1038/s41598-020-71158-9 PMC7455712 32859971

[bb81] UMPA (2017). *UMPA*, https://github.com/pierrethibault/umpa/ [accessed on 15 June 2023].

[bb83] Wang, H., Kashyap, Y. & Sawhney, K. (2015). *Opt. Express*, **23**, 23310–23317. 10.1364/OE.23.023310 26368432

[bb778] Weitkamp, T., Diaz, A., David, C., Pfeiffer, F., Stampanoni, M., Cloetens, P. & Ziegler, E. (2005). *Opt. Express*, **13**, 6296. 10.1364/opex.13.006296 19498642

[bb84] Weitkamp, T., Haas, D., Wegrzynek, D. & Rack, A. (2011). *J. Synchrotron Rad.* **18**, 617–629. 10.1107/S0909049511002895 21685680

[bb85] Westneat, M. W., Socha, J. J. & Lee, W.-K. (2008). *Annu. Rev. Physiol.* **70**, 119–142. 10.1146/annurev.physiol.70.113006.100434 18271748

[bb86] Wilkins, S. W., Gureyev, T. E., Gao, D., Pogany, A. & Stevenson, A. W. (1996). *Nature*, **384**, 335–338.

[bb87] WXSVT (2020). *WaveletSpeckleTracking*, https://github.com/APS-XSD-OPT-Group/WaveletSpeckleTracking/ [accessed on 15 June 2023].

[bb88] Zanette, I., Zhou, T., Burvall, A., Lundström, U., Larsson, D., Zdora, M., Thibault, P., Pfeiffer, F. & Hertz, H. (2014). *Phys. Rev. Lett.* **112**, 253903. 10.1103/PhysRevLett.112.253903 25014818

[bb90] Zdora, M., Thibault, P., Zhou, T., Koch, F. J., Romell, J., Sala, S., Last, A., Rau, C. & Zanette, I. (2017). *Phys. Rev. Lett.* **118**, 203903. 10.1103/PhysRevLett.118.203903 28581800

[bb89] Zdora, M.-C. (2018). *J. Imaging*, **4**, 60.

[bb91] Zuo, C., Li, J., Sun, J., Fan, Y., Zhang, J., Lu, L., Zhang, R., Wang, B., Huang, L. & Chen, Q. (2020). *Opt. Lasers Eng.* **135**, 106187.

